# Significant improvement of olfactory performance in sleep apnea patients after three months of nasal CPAP therapy – Observational study and randomized trial

**DOI:** 10.1371/journal.pone.0171087

**Published:** 2017-02-03

**Authors:** Bettina Boerner, Gabrielo M. Tini, Patrick Fachinger, Sereina M. Graber, Sarosh Irani

**Affiliations:** 1 Clinic of Pulmonary and Sleep Medicine, Cantonal Hospital Aarau, Aarau, Switzerland; 2 Anthropological Institute and Museum, University of Zürich-Irchel, Zürich, Switzerland; Associazione OASI Maria SS, ITALY

## Abstract

**Objectives:**

The olfactory function highly impacts quality of life (QoL). Continuous positive airway pressure is an effective treatment for obstructive sleep apnea (OSA) and is often applied by nasal masks (nCPAP). The influence of nCPAP on the olfactory performance of OSA patients is unknown. The aim of this study was to assess the sense of smell before initiation of nCPAP and after three months treatment, in moderate and severe OSA patients.

**Methods:**

The sense of smell was assessed in 35 patients suffering from daytime sleepiness and moderate to severe OSA (apnea/hypopnea index ≥ 15/h), with the aid of a validated test battery (Sniffin’ Sticks) before initiation of nCPAP therapy and after three months of treatment. Additionally, adherent subjects were included in a double-blind randomized three weeks CPAP-withdrawal trial (sub-therapeutic CPAP pressure).

**Results:**

Twenty five of the 35 patients used the nCPAP therapy for more than four hours per night, and for more than 70% of nights (adherent group). The olfactory performance of these patients improved significantly (p = 0.007) after three months of nCPAP therapy. When considering the entire group of patients, olfaction also improved significantly (p = 0.001). In the randomized phase the sense of smell of six patients deteriorated under sub-therapeutic CPAP pressure (p = 0.046) whereas five patients in the maintenance CPAP group showed no significant difference (p = 0.501).

**Conclusions:**

Olfactory performance improved significantly after three months of nCPAP therapy in patients suffering from moderate and severe OSA. It seems that this effect of nCPAP is reversible under sub-therapeutic CPAP pressure.

**Trial registration:**

ISRCTN11128866

## Introduction

Obstructive sleep apnea (OSA) is characterized by repetitive upper airway obstruction during sleep and consecutive arousals. Continuous positive airway pressure (CPAP) therapy has been shown to efficiently treat OSA and to improve quality of life (QoL) mainly by decreasing symptoms of excessive sleepiness [1]. In most cases CPAP therapy is applied with the aid of nasal masks (nCPAP). The clinical observation that nCPAP is frequently associated with irritation of the nasal mucosa is supported by scientific data, which show increased inflammatory activity of the nasal mucosa after initiation of nCPAP therapy [2,3]. Furthermore, OSA itself seems to be associated with decreased olfactory functions [4]. On the other hand recent data show that regular CPAP use can revert nasal inflammation in OSA patients [5].

In accordance with other observations [6–8] our group showed that better sense of smell is associated with increased QoL [9].

The impact of nCPAP therapy on olfactory performance is unknown. Therefore, the rationale of this longitudinal study was to investigate the olfactory performance of patients suffering from moderate to severe OSA, before initiation of nCPAP therapy and after three months of treatment, in consecutive outpatients of our department of pulmonary and sleep medicine.

## Materials and methods

Consecutive outpatients of our department of pulmonary and sleep medicine were included in the current study if they were ≥ 18 years of age, suffered from daytime sleepiness and showed moderate to severe OSA (apnea hypopnea index (AHI) ≥ 15 per hour) in a respiratory sleep study. Exclusion criteria were as follows: (1) lack of written informed consent, (2) insufficient knowledge of the German language, (3) known disease of the nasal cavity or sinuses, or topical nasal therapy usage.

Study participants were recruited between November 19^th^ 2014 and July 15^th^ 2015. All patients were referred from General Practitioners, as a consequence of excessive sleepiness and snoring. In addition to patient’ history and a physical examination, an overnight respiratory sleep study was obtained. The parameters monitored contained non-stop recordings from finger oxygen saturation, body position, chest and abdominal motion and nose airflow. The analysis of the respiratory sleep study was performed by experienced sleep medical consultants, according to current guidelines [10]. Daytime sleepiness was assessed with the aid of a validated German version of the Epworth sleepiness scale [11].

Sense of smell was tested with the aid of the validated [12,13] Sniffin’ Sticks battery (Burghart, Wedel, Germany). The test was performed bi-rhinally, which takes 30 to 40 minutes. The procedure is described in detail elsewhere [12], briefly, the test battery contains 112 odour-dispensing pens to evaluate odour threshold, odour discrimination and odour identification. The results of the odour threshold, discrimination and identification are summarized in an overall “TDI-Score”. Normosmia (TDI ≥ 31), hyposmia (15 < TDI < 31) and anosmia (TDI ≤ 15) have been defined in a large normative population [14]. Additionally, patients were asked to self-estimate their sense of smell on a visual analogue scale (VAS) of 0 to 10.

nCPAP treatment commenced as standard of care treatment with autotitrating devices (pressure 4 to 12 cm H_2_O, AirSense 10 AutoSet, ResMed, Ltd., Bella Vista, Australia) and the patients were encouraged to use a heated humidification system (HumidAir, ResMed, Ltd., Bella Vista, Australia). The auto pressure modus was used during the whole study period. A nasal mask (Mirage FX, ResMed, Ltd., Bella Vista, Australia) was used with all patients. After initiation of nCPAP therapy by a trained study nurse, further patient support was achieved with the aid of a web-based telemedicine system (AirView, ResMed, Ltd., Bella Vista, Australia). The patients’ telemedicine data was screened for poor adherence, high mask leakages or residual apneas by a specially trained study nurse on a weekly basis and telephone contact was established if necessary. The first follow-up study visit was scheduled three months after initiation of nCPAP treatment. Therapy adherence was defined as use of nCPAP ≥ 4 hours per night during ≥ 70% of the recorded nights [15]. At the moment of the assessment of the sense of smell after three months the investigator was unaware of patients’ therapy adherence.

After three months of nCPAP therapy adherent patients were asked by telephone to take part in a randomized three weeks CPAP-withdrawal trial. Recruitment for this part of the study took place between July 5^th^ 2016 and July 12^th^ 2016. During this part of the study nCPAP therapy was set on a sub-therapeutic level (four centimeters of water) for the period of three weeks with the aid of telemedicine in a blinded and randomized fashion (block randomization, performed by SI, blinded for study nurse and participants). TDI score was re-assessed thereafter by a blinded investigator (BB). TDI score after three weeks was compared with the TDI value after three months of nCPAP therapy in in the therapy maintenance group and the therapy withdrawal group, respectively.

Sample size: in an earlier study [9] of a population of lung patients we found a mean TDI score of 29, the standard deviation of the mean TDI score was six. Considering a change in the TDI score of four or more to be of clinical relevance [16] according to Lehr [17] a sample size of 30 patients was regarded to be appropriate (power 80%, level of significance 0.05).

Statistica, Version 10.0 (StatSoft, Inc., Tulsa, USA) was used for statistical analyses. Absolute numbers, percent, median and interquartile range were used for descriptive data, the non-parametric Wilcoxon matched pair test was used to compare the measured parameters with baseline data after three months treatment. The correlation of self-estimated olfactory function and TDI score and mean daily use (per total days) and change of TDI-score after three months, respectively, were performed with the aid of the non-parametric Spearman rank test. p < 0.05 was considered statistically significant.

The study protocol was approved by the Ethics Committee of the Nordwest- und Zentralschweiz, EKNZ, Switzerland (EKNZ 2014–335). Written informed consent was obtained from all patients. Written informed consent was obtained from all patients. For the randomized part an amendment to the initial protocol was approved by the Ethics Committee of the Nordwest- und Zentralschweiz as well. A separate written informed consent was obtained from all eleven participants of the randomized part of the study.

This trial was retrospectively registered (ISRCTN11128866) since at the beginning it was intended to be rather an observational study than a clinical trial. The authors confirm that all ongoing and related trials for this intervention are registered.

## Results

**Fig 1:**
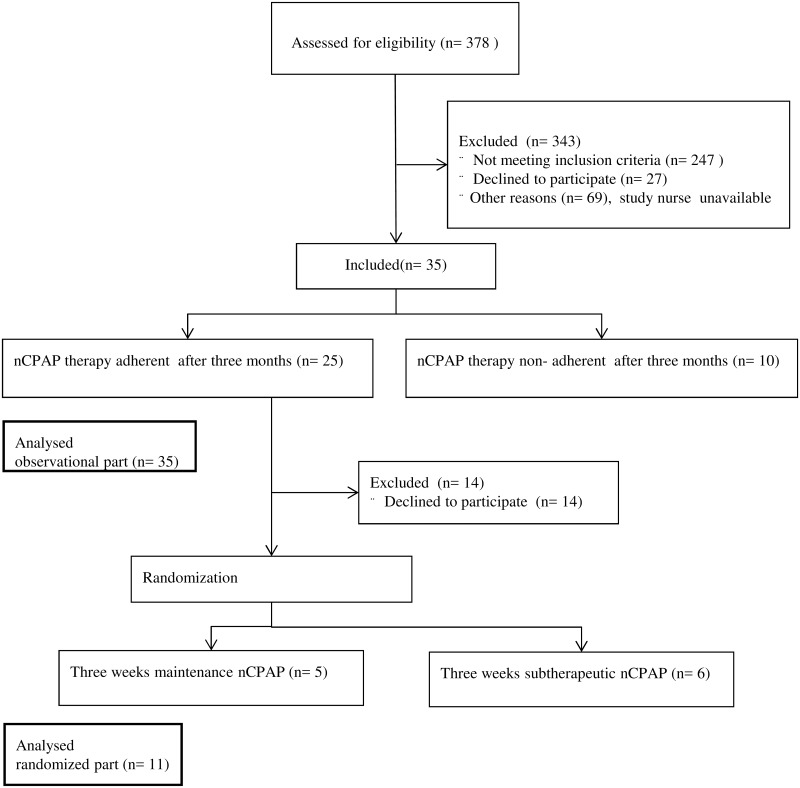
Flowchart of the trial.

Thirty five consecutive patients suffering from moderate to severe OSA were included in the current longitudinal study. The flowchart of the trial is shown in Fig 1.

Due to poor adherence and complained discomfort in twelve patients (34%) a change to another nasal mask was realized within the first two weeks. Despite this measure the therapy adherence remained insufficient in ten of these patients (29%), according to the pre-defined criterion. Therefore, further analyses were performed separately. The patient baseline data is summarized in Table 1. The study subjects’ CPAP adherence data is shown in Table 2.

**Table 1:** Demographic and clinical data of the study population.

	Entire group	Adherent*	Non-adherent*
Baseline characteristics	n = 35	n = 25	n = 10
Age, years	53 (44 to 60)	53 (51 to 60)	50 (44 to 59)
Gender, female	4 (11)	2 (8)	2 (20)
BMI, kg/m2	36 (33 to 40)	33.7 (30.8 to 40)	31.6 (23 to 36.1)
Current smoker	10 (28)	5 (20)	5 (50)
AHI, per hour	55 (36 to 70)	63 (38 to 76)	40 (36 to 55)
AHI ≥ 30	32 (91)	23 (92)	9 (90)
30 > AHI ≥ 15	3 (9)	2 (8)	1 (10)
SaO2 drop ≥4%, events/hour	58 (39 to 69)	64 (40 to 78)	46 (39 to 50)
ESS score	9 (7 to 11)	9 (7 to 10)	9 (7 to 15)
Comorbidity			
Ischaemic heart disease	4 (11)	3 (12)	1 (10)
Hypertension	16 (46)	11 (44)	5 (50)
Diabetes mellitus	6 (17)	5 (20)	1 (10)
Peripheral vascular disease	1 (3)	0 (0)	1 (10)
Cerebral vascular disease	0 (0)	0 (0)	0 (0)

BMI: body mass index, AHI: apnea-hypopnea index; ESS: Epworth sleepiness scale; disease,

*adherence: in 70% of the nights at least 4 hours CPAP use per night.

Data presented as median (IQR) or number (%).

**Table 2:** nCPAP adherence data.

	Adherent*	Non-adherent*
	(n = 25)	(n = 10)
Percentage of days on which nCPAP was used, %	99 (98 to 100)	76 (72 to 83)
Mean daily use of nCPAP per total days, hours	6.3 (5.7 to 6.9)	3.2 (2.9 to 3.4)
Mean daily use of nCPAP per day used, hours	6.5 (5.8 to 6.9)	4.3 (3.5 to 5.4)
Percentage of days on which nCPAP was used for > 4h	97 (88 to 98)	44 (35 to 49)

nCPAP: nasal continuous positive airway pressure,

*adherence: in 70% of the nights at least 4 hours CPAP use per night.

Data presented as median (IQR).

**Fig 2:**
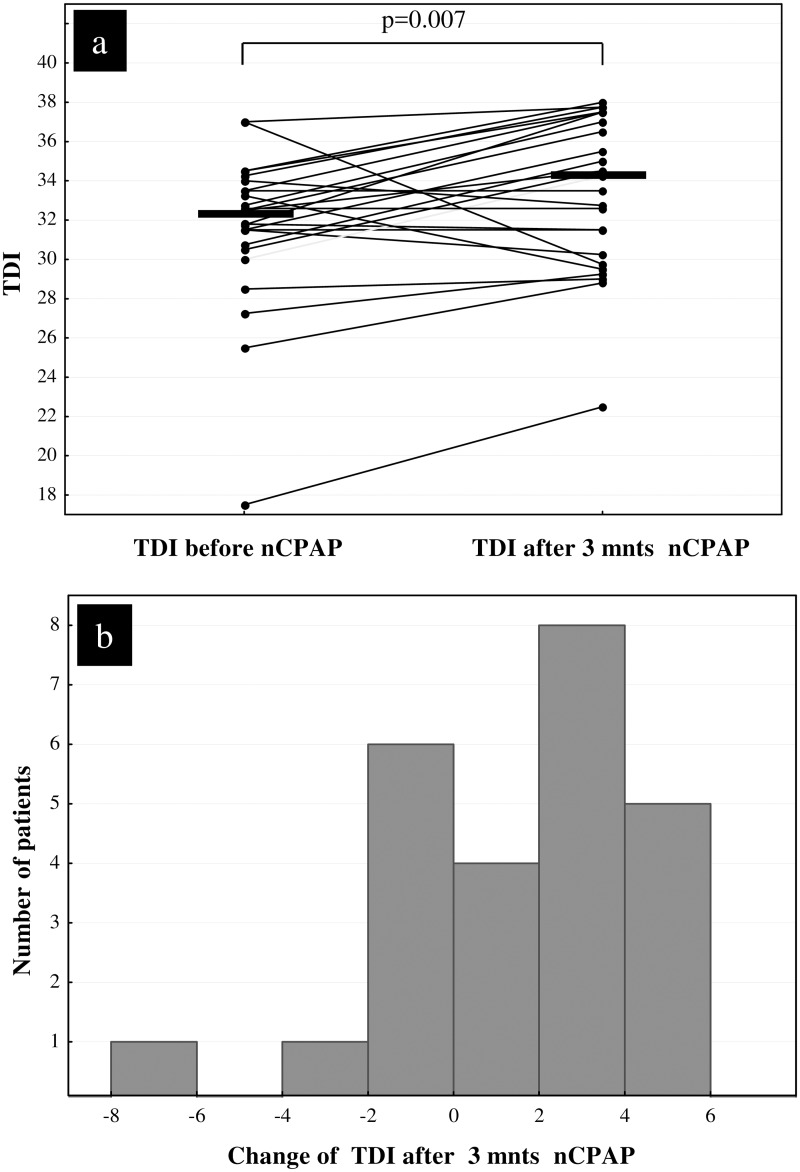
Case profiles (a) and histogram of change of TDI (b) after three months (mnts) of nasal CPAP therapy in 25 adherent sleep apnea patients. TDI: threshold, discrimination and identification overall score, nCPAP: nasal continuous positive airway pressure.

Subjective and objective measures of olfactory performance and sleepiness data of the entire group and the adherent patients, respectively, at baseline and after three months of nCPAP therapy is summarized and compared in Table 3. No significant correlation between AHI and olfactory function was seen at the initial assessment (data not shown). Values for odour threshold, odour discrimination and odour identification of the ten non- adherent patients at baseline and after three months, respectively, were 8.2 (6.5 to 9.2), 12.5 (12 to 14), 12 (12 to 15) and 8.5 (7.2 to 10), 13.5 (12 to 14), 12.5 (12 to 14), respectively. There was also an improvement in all odour qualities in the non-adherent patients however the change did not reach statistical significance. The TDI scores of the ten non-adherent patients were 33.7 (32 to 34.5) at baseline and 34.9 (33 to 35.5) after three months. In this summarized score statistical significance was also not reached (p = 0.109). The TDI score case profiles and the histogram of TDI score change of the 25 adherent patients are shown in Fig 2. Improvement in TDI-score of ≥ 4 was observed in 9 of the 25 adherent patients (36%) and 2 of the 10 non-adherent patients (20%). In the 35 patients, the correlation of self-estimated olfactory performance and TDI score was poor (at baseline R = 0.057, p = 0.743 and after three months R = 0.249, p = 0.148, respectively). No statistically significant correlation of change of TDI-score after three months and mean daily use of nCPAP per total days was found (R = 0.25, p = 0.141).

**Table 3:** Olfactory performance and sleepiness before and after nCPAP treatment of the entire group (n = 35) and of the 25 therapy adherent* sleep apnea patients.

	Baseline	After three months of CPAP	p
Entire group	n = 35	n = 35	
Odor treshold	7.7 (7 to 9)	8.7 (7.5 to 10.2)	0.041
Odor discrimination	12 (11 to 13)	12 (11 to 14)	0.353
Odor identification	12 (11 to 14)	13 (12 to 14)	0.101
TDI-score	32.5 (30.7 to 34)	34.5 (31.5. to 36.5)	0.001
Sense of smell self estimation VAS	6.7 (5 to 7)	7 (5.5 to 8)	0.109
ESS	9 (7 to 11)	4.5 (3 to 7)	<0.001
Adherent patients	n = 25	n = 25	
Odor treshold	7.7 (7.5 to 8.5)	9 (7.7 to 10.2)	0.058
Odor discrimination	12 (11 to 13)	12 (11 to 14)	0.635
Odor identification	12 (11 to 14)	13 (12 to 14)	0.055
TDI-score	32.5 (30.7 to 33.5)	34.2 (30.2 to 37)	0.007
Sense of smell self estimation VAS	6.5 (6 to 7)	6.5 (6 to 7)	0.034
ESS	9 (7 to 10.5)	5 (3 to 7)	<0.001

*adherence: in 70% of the nights at least 4 hours CPAP use per night.

TDI: threshold, discrimination and identification overall score, VAS: visual analog scale (0–10), ESS: Epworth sleepiness scale, Data presented as median (IQR), Wilcoxon matched pair test was used

**Fig 3:**
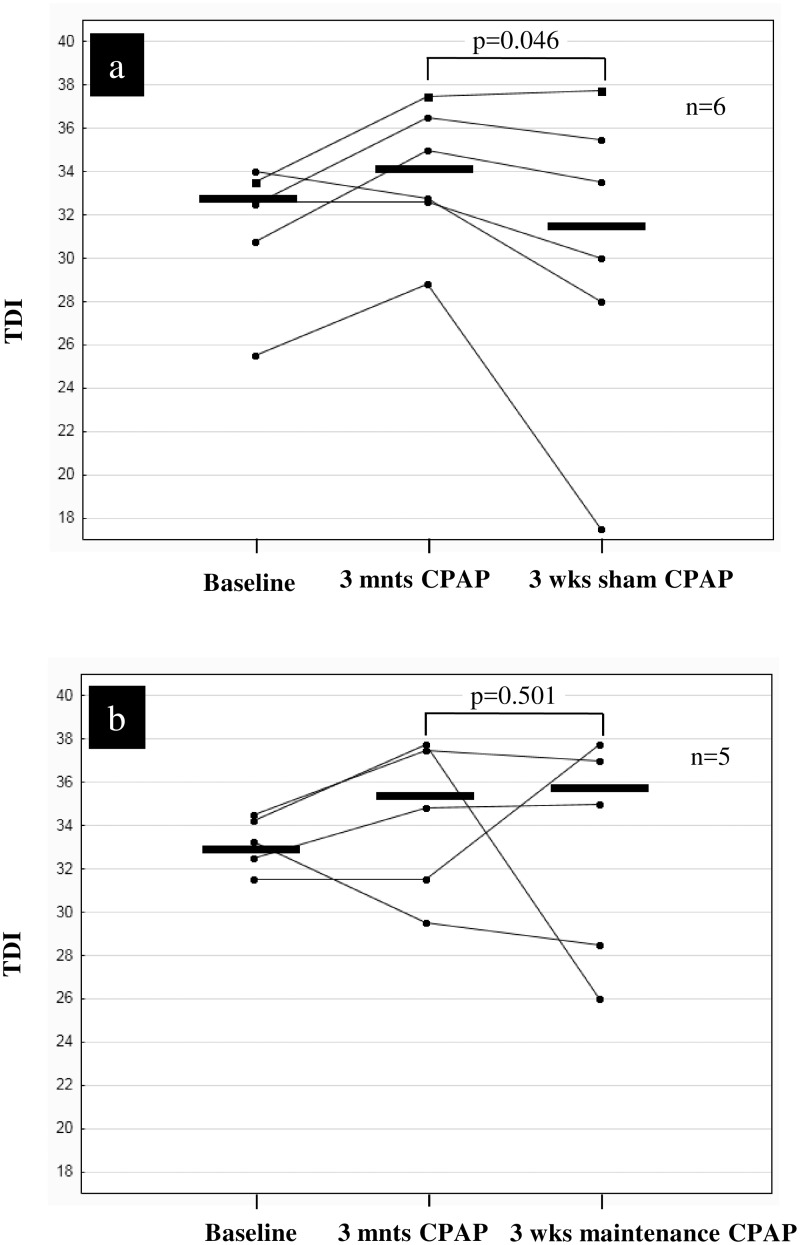
Olfactory performance at baseline, three months (mnts) after start of nasal CPAP therapy and during the three weeks (wks) blinded, randomized therapy withdrawal trial in 11 adherent sleep apnea patients (Panel a: therapy withdrawal group, panel b: therapy maintenance group). TDI: threshold, discrimination and identification overall score.

Eleven out of the 25 adherent patients agreed to take part in the randomized three weeks CPAP-withdrawal trial. TDI case profiles of the six therapy withdrawal patients and the five therapy maintenance patients are graphically shown in Fig 3.

No side effects or harms were reported.

## Discussion

Although still underestimated in the current research, sense of smell is important and impacts quality of life. However, whether CPAP therapy influences sense of smell is not known. The aim of the current study was to investigate the sense of smell in patients suffering from moderate to severe sleep apnea before initiation of nasal CPAP therapy and after three months treatment.

Our finding that olfaction significantly improves in patients suffering from moderate and severe sleep apnea after three months of nasal CPAP therapy is remarkable in several ways.

Olfactory performance significantly improved after three months of nCPAP therapy in adherent patients when the TDI-score as an objective measure of this complex neurosensory system is considered. The predefined, clinically significant improvement of TDI-score of four or more points was achieved in 36 percent of the patients in this group. Although not statistically significant, scores for odour threshold and odour identification also improved after three months. The odour discrimination score seems to be affected to a lesser extent. In a recent cross-sectional study Salihoglu et al. [4] showed a strong negative correlation between AHI and odour performance in 75 subjects. We cannot confirm this observation, which might be the consequence of our relatively small group of patients. However, our observation of significant and clinically relevant improvement of the sense of smell after nCPAP treatment fits quite well with the hypothesis, that sleep apnea negatively impacts sense of smell in affected subjects. Whether this is the consequence of local negative effects of sleep apnea on the nasal structures [18] or the consequence of the multiple and well known negative impacts of sleep apnea on neurocognitive functions [19] remains speculative. Considering the fact that nCPAP therapy itself induces nasal mucosal inflammation to some degree [2,3] with potentially negative consequences on sense of smell the later hypothesis seems more likely in our opinion. On the other hand recent data showed that CPAP therapy is associated with decreased local inflammatory cell accumulation [5] which could argue also for a local benefit of the nasal mucosa.

The fact that the improvement of the olfactory performance was reversible to a certain degree in most patients after three weeks of sham CPAP therapy supports the interpretation that OSA negatively impacts sense of smell. Whereas the olfactory performance in most patients of the therapy maintenance group remained unchanged one patient further improved his TDI compared to the TDI at three months. Whether in some cases the positive effects of nCPAP occur after a longer than three months treatment period remain speculative.

In contrast to other clinical studies, in which objective improvement of olfactory performance was not perceived by the patients [9,20] the subjective perception of the sense of smell in our population was significantly improved in the nCPAP adherent population. Since our study had no control group this result should be interpreted with caution. We cannot rule out patients’ perception to be influenced by spectrum bias and observer bias. However, the fact that the sniffing’ sticks battery test is complex, well validated and hard to influence subjectively neither patients nor investigator are likely to have biased the current study results. This is particularly true since during the second test after three months of nCPAP therapy the investigator was not aware of the patients’ therapy adherence.

The predefined and well established definition of nCPAP therapy adherence we adopted in the current study is somewhat arbitrary. Although we were not able to show a significant correlation between length of daily nCPAP use and improvement of olfactory performance, a clinically relevant improvement of the TDI-score of ≥4 points was observed more frequently in adherent patients (36 versus 20 percent of patients). Greater improvement of OSA symptoms has been shown to be related to increased duration of CPAP usage in several studies [21,22]. On the other hand even short daily usage of CPAP can provide some clinical benefits [21,23]. We are unable to answer the question whether or not a dose response of nCPAP therapy exists regarding improvement of the sense of smell in OSA patients with the current data. This is the consequence of the relatively small group of patients and the relatively high therapy adherence even in the non-adherent group.

This study has several limitations. We did not have a control group of untreated OSA patients. For ethical reasons we feel it is impossible to withhold CPAP from symptomatic patients suffering from moderate to severe OSA for three months.

Another shortcoming is the relatively small group of subjects. On the other hand it is remarkable that we were able to find consistent, significant and clinically relevant results even in this limited number of patients.

In summary, in the current preliminary longitudinal study we have shown that the sense of smell significantly improves after three months of nCPAP therapy in patients suffering from moderate to severe sleep apnea. This is particularly true when the therapy adherence is high.

Additionally, we found evidence, that this positive effect is reversible to a certain degree after three weeks of sub-therapeutic CPAP therapy.

## Supporting information

S1 File Registration details. ISRCTN Study record 32654.
(PDF)

S2 File Consort checklist. Consort 2010 Checklist.
(DOC)

S3 File Trial protocol original. Original Protocol in German.
(DOCX)

S4 File Trial protocol translated. Protocol translated in English.
(DOCX)

S5 File Details of approval. Details of Study approval.
(PDF)

S6 File Details of approval amendment. Approval of amendment.
(PDF)
