# Supplementary material for: Significant improvement of olfactory performance in sleep apnea patients after three months of nasal CPAP therapy – Observational study and randomized trial
Source: PLoS One. 2017 Feb 3;12(2):e0171087. doi: 10.1371/journal.pone.0171087 (PMC5291379; doi:10.1371/journal.pone.0171087)
Supplement: S1 File — ISRCTN Study record 32654. (PDF) [file pone.0171087.s001.pdf]

# Study record 32654

*Generated: 04/10/2016 19:06:45*
*Editorial Status: Submitted*

## Title and Additional Identifiers

### Submission number
32654

### ISRCTN

### DOI

### Public title
Improved olfaction under CPAP therapy

### Scientific title
Significant improvement of olfactory performance in sleep apnea patients after three months of nasal CPAP therapy

### Acronym

### EudraCT number

### ClinicalTrials.gov number

### Protocol /serial number
EKNZ 2014-335

### Condition category

### Date Applied
04/10/2016

### Date Assigned

### Last Edited
04/10/2016

### Prospective/Retrospective

### Overall Trial Status
Completed

### Recruitment Status
No longer recruiting

## Study Information

## Study hypothesis
The influence of nasal CPAP on the olfactory performance of OSA patients is unknown. The aim of this study was to assess the sense of smell before initiation of nasal CPAP and after three months treatment, in moderate and severe OSA patients.

## Ethics approval
The study protocol was approved by the Ethics Committee of the Nordwest- und Zentralschweiz, EKNZ, Switzerland (EKNZ 2014-335) on November 18th 2014. Written informed consent was obtained from all patients. Written informed consent was obtained from all patients. For the randomized part an amendment to the initial protocol was approved by the Ethics Committee of the Nordwest- und Zentralschweiz on May 19th 2016 as well. A separate written informed consent was obtained from all eleven participants of the randomized part of the study.

## Study design
The aim of this study was to assess the sense of smell before initiation of nCPAP and after three months treatment, in moderate and severe OSA patients (observational part). After three months of nCPAP therapy adherent patients were asked by telephone to take part in a randomized three weeks CPAP-withdrawal trial (interventional part).

## Primary study design
Observational

## Secondary study design
Secondary study part was a randomized CPAP therapy withdrawal part

## Trial setting
Hospitals

## Trial type
Treatment

## Overall trial start date
19/11/2014

## Overall trial end date
16/08/2016

## Overall trial status override

## Reason abandoned

## Condition
Olfactory performance in moderate and severe sleep apnea patients

## Interventions
Nasal continuous positive airway pressure for three months

## Intervention Type
Device

**Phase**

**Drug name(s)**

**Primary outcome measures**
The sense of smell was assessed in patients suffering from daytime sleepiness and moderate to severe OSA (apnea/hypopnea index ≥ 15/h), with the aid of a validated test battery (Sniffin' Sticks) before initiation of nCPAP therapy and after three months of treatment.

**Secondary outcome measures**
Adherent subjects were included in a double-blind randomized three weeks CPAP-withdrawal trial (sub-therapeutic CPAP pressure).

**Trial website**

**Participant information sheet**

# Eligibility

**Participant inclusion criteria**
1. outpatients of our department of pulmonary and sleep medicine
2. ≥ 18 years of age
3. suffer from daytime sleepiness
4. show moderate to severe OSA (apnea hypopnea index (AHI) ≥ 15 per hour) in a respiratory sleep study.

**Participant type**
Patient

**Age group**
Adult

**Gender**
Both

**Target number of participants**
30 patients

**Participant exclusion criteria**
1. lack of written informed consent
2. insufficient knowledge of the German language
3. known disease of the nasal cavity or sinuses, or topical nasal therapy usage

**Recruitment start date**
19/11/2014

**Recruitment end date**
15/07/2015

**Recruitment status override**

# Locations

### Countries of recruitment
Switzerland

## Trial participating centres

## Trial Centre

### Trial Centre Name
Clinic of Pulmonary and Sleep Medicine, Cantonal Hospital Aarau

### Address
Tellstrasse

### City
Aarau

### Country
Switzerland

### Zip
CH-5001

# Plain English Summary

The olfactory function highly impacts quality of life (QoL). Continuous positive airway pressure is an effective treatment for obstructive sleep apnea (OSA) and is often applied by nasal masks ( nCPAP). The influence of nCPAP on the olfactory performance of OSA patients is unknown. The aim of this study was to assess the sense of smell before initiation of nCPAP and after three months treatment, in moderate and severe OSA patients.
The sense of smell was assessed in 35 patients suffering from daytime sleepiness and moderate to severe OSA (apnea/hypopnea index ≥ 15/h), with the aid of a validated test battery (Sniffin' Sticks) before initiation of nCPAP therapy and after three months of treatment. Additionally, adherent subjects were included in a double-blind randomized three weeks CPAP-withdrawal trial (sub-therapeutic CPAP pressure).
Twenty five of the 35 patients used the nCPAP therapy for more than four hours per night, and for more than 70% of nights (adherent group). The olfactory performance of these patients improved significantly (p=0.007) after three months of nCPAP therapy. When considering the entire group of patients, olfaction also improved significantly (p=0.001). In the randomized phase the sense of smell of six patients deteriorated under sub-therapeutic CPAP pressure (p=0.046) whereas five patients in the maintenance CPAP group showed no significant difference (p=0.501) .
Olfactory performance improved significantly after three months of nCPAP therapy in patients suffering from moderate and severe OSA. It seems that this effect of nCPAP is reversible under sub-therapeutic CPAP pressure.

# Results and Publications

**Publication and dissemination plan**

**Intention to publish date**

**Participant level data**
Not expected to be available

**Results - basic reporting**

**Results – Plain English Summary**

**Publication summary**

# Publication citation(s)

# Contact(s)

## Contact


**Type**
Scientific

**Title**
Dr

**Name**
Sarosh Irani

**ORCID ID**

**Address**
Clinic of Pulmonary and Sleep Medicine, Cantonal Hospital Aarau

**City**
Aarau

**Country**
Switzerland

**Zip**
CH-5001

**Tel**
+41 62 838 44 70

**Email**
siran@gmx.ch

**Privacy**
Public

# Sponsor(s)

## Sponsor

**Organisation**
Cantonal Hospital Aarau

**Address**
Tellstrasse

**City**
Aarau

**Country**
Switzerland

**Zip**
CH-8057

**Tel**
+41 62 838 44 72

**Email**
pneumologie@ksa.ch

**Type**
Hospital/treatment centre

**Website**

**Privacy**
Public

# Funder(s)

**Funding Type**
Not defined

## Funder

**Funder Name**
: The Research Funding from the Research Council of the Cantonal Hospital Aarau, Switzerland provided financial support in the form of funding of the salary of the study nurse (60’000 Euro).

**Alternative Name(s)**

**Funding Body Type**

**Funding Body Subtype**

**Location**

## Applicant Details

**Name**
Sarosh Irani

**ORCID ID**

**Address**

**City**

**Country**

**Zip**

**Tel**

**Email**
siran@gmx.ch

## Payment Method

**Payment method**
Online payment

**Trusted funder**

## Invoice Details

**Name**

**Institution**

**Address**

**City**

**State**

**Country**

**Zip**

Email

Purchase order/ reference number

VAT number

Why did you choose ISRCTN to register your trial?
