# Supplementary material for: Significant improvement of olfactory performance in sleep apnea patients after three months of nasal CPAP therapy – Observational study and randomized trial
Source: PLoS One. 2017 Feb 3;12(2):e0171087. doi: 10.1371/journal.pone.0171087 (PMC5291379; doi:10.1371/journal.pone.0171087)
Supplement: S3 File — Original Protocol in German. (DOCX) [file pone.0171087.s003.docx]

Clinical Study Protocol

Change of olfactory performance after initiation of CPAP therapy in sleep apnea: a longitudinal study

This document is the Clinical Protocol template for IIT (Investigator initiated Trials) studies. AGEK strongly recommends using this template to develop clinical research protocols for trials testing an investigational medicinal product (IMP) or a medical device (MD) to be submitted to Swiss authorities.

This template is suitable for studies:

- involving IIT,
- performed in Switzerland, respectively where the Sponsor-Investigator is located in Switzerland
- where the study question does relate to the use of drug(s) or medical device effect(s),
- where the Swiss law on therapeutic products (HMG/LPTh and Federal Act on Medicinal Products and Medical Devices) applies,
- where the Swiss law on human research (Federal Act on Research involving Human Beings (HRA)) and its applicable ordinance ClinO/KlinV/OClin applies,
- that are interventional*

*health related interventional studies include research in preventive, diagnostic, therapeutic, palliative or rehabilitation activities that are examined in the context of a clinical trial.

The current template is based on:

- [AGEK – CT CER](http://www.swissethics.ch/templates.html) / [Swissmedic](http://www.swissmedic.ch/bewilligungen/00089/00282/index.html?lang=de) guidelines: “Studienprotokolle von klinischen‚ Investigator-initiated’ Studien/Versuchen / Exigences des protocoles d’études/d’essais cliniques initiés par l’investigateur” dated 24.02.2009,
- the Federal Act on Research involving Human Beings ([HRA](http://www.admin.ch/ch/e/rs/8/810.30.en.pdf)) and its applicable ordinance ([ClinO](http://www.admin.ch/ch/e/rs/8/810.305.en.pdf)/[Klin](http://www.admin.ch/opc/de/official-compilation/2013/3407.pdf)V/[OClin](http://www.admin.ch/opc/fr/official-compilation/2013/3407.pdf))
- the [SPIRIT statement](http://www.spirit-statement.org) and
- [ICH-GCP E6](http://www.ich.org/fileadmin/Public_Web_Site/ICH_Products/Guidelines/Efficacy/E6_R1/Step4/E6_R1__Guideline.pdf), section 6
- [EN ISO14155:2011](https://www.iso.org/obp/ui/#iso:std:iso:14155:ed-2:v1:en): Annex A
- Swiss clinical trials portal (http://www.kofam.ch/en/swiss-clinical-trials-portal.html)

This template attempts to provide a general format applicable to all clinical trials evaluating an investigational product (drugs or medical devices).

Note that *instructions* are indicated in *blue italics* and they need be deleted (or alternatively may be formatted as “hidden Text” that will not show in printing).

Section headings and template text formatted in **regular type red** gives you reference to the legal requirements. This text may be deleted.

Section headings and template text formatted in regular type (black) should be included in your protocol document as provided in the template.

Header and footer should contain the following information (on all pages): [Protocol Title], [Page x of xx], [version x, DD/MM/YYYY], [Study ID]

In places where the information is redundant, it is acceptable to reference another section, to document or to state its redundancy but the section has not to be deleted.

Refer questions regarding use of this protocol template to swissethics/AGEK, [info@swissethics.ch](mailto:info@swissethics.ch), phone: +41 (41) 440 26 67, www.swissethics.ch.

This template was developed by a task force initiated by the Federal Office of Public Health (FOPH) and the AGEK / CT CER during 2013 and under the lead and coordination of the Swiss Clinical Trial Organisation (SCTO), Basel. Clinical research experts from 8 institutions reviewed the template. The FOPH and AGEK reviewed the template and recommend its use.

Members of the task force no. 6 and contributors to this template:

- FOPH, Dr Andri Christen
- AGEK, Dr Wolfgang Tschacher
- CTC Zurich, Dr Eva Brombacher
- CTU St Gallen, Roger Getzmann
- CTC Lausanne and Cochrane Switzerland, Dr Erik von Elm
- CTC Geneva, Dr Christophe Combescure
- EOC Ticino, Dr Liliane Petrini
- SAKK, Dr Christiane Pilop
- SCTO, Annette Magnin and Dr Caecilia Schmid

Reviewers:

- EOC TI, Dr Claudio Gobbi
- EOC TI, Dr Mauro Manconi
- HUG: Prof. Dr Bernard Hirschel
- HUG / EC: Dr Sandrine Charvat
- Inselspital / Uni. Bern, Prof. Dr Peter Jüni, Dr Sven Trelle
- KSSG, Prof. Dr Christoph Driessen
- SAKK, Dr Pirus Ghadjar (Charité Berlin)
- SPZ / EC Luzern, Dr Angela Frotzler
- USZ, PD Dr Christian Baumann
- USZ, Dr Cédric Poyet
- Scienceindustries, Dr Daniela Gunz

<<Protocol template: Interventional study with investigational medicinal product (IMP) / medical device (MD)>>

Clinical Study Protocol

INSERT TITLE OF THE PROTOCOL

Change of olfactory performance after initiation of CPAP therapy in sleep apnea: a longitudinal study (AGEK 1; SPIRIT #1)

SHORT TITLE : Olfactory performance after initiation of CPAP therapy

| Study Type: | Longitudianl study |
| --- | --- |
| Study Categorisation: | Kategorie A |
| Study Registration: | N.A. |
| Study Identifier: | Kantonsspital Aarau AG |
| Sponsor, Sponsor-Investigator or Principal Investigator: | PD Dr.med. S.Irani, Pneumologie und Schlafmedizin, Tellstrasse, Kantonsspital Aarau |
| Investigational Product: | N.A. |
| Protocol Version and Date: | Version 1; 08.08.2014 |

CONFIDENTIAL

The information contained in this document is confidential and the property of the sponsor. The information may not - in full or in part - be transmitted, reproduced, published, or disclosed to others than the applicable Competent Ethics Committee(s) and Regulatory Authority(ies) without prior written authorisation from the sponsor except to the extent necessary to obtain informed consent from those who will participate in the study.

Signature Page(s)

(AGEK 1.1; ICH E6 6.1)

ICH E6: Have signature pages with name and title of the person(s) authorised to sign the protocol and the protocol amendment(s) for the sponsor or of the medical expert (if applicable), the investigator responsible for conducting the trial, the statistician (if applicable)

| Study number | N.A. |
| --- | --- |
| Study Title | Change of olfactory performance after intitiation of CPAP therapy in sleep apnea: a longitudinal study |

The Sponsor-Investigator and trial statistician have approved the protocol version 01 (08.08.2014), and confirm hereby to conduct the study according to the protocol, current version of the World Medical Association Declaration of Helsinki, ICH-GCP guidelines or ISO 14155 norm if applicable and the local legally applicable requirements.

Sponsor-Investigator: PD. Dr.med. S.Irani

| Place/Date |  | Signature |
| --- | --- | --- |

I have read and understood this trial protocol and agree to conduct the trial as set out in this study protocol, the current version of the World Medical Association Declaration of Helsinki, ICH-GCP guidelines or ISO 14155 norm and the local legally applicable requirements.

| Site | Kantonsspital Aarau, Pneumologie und Schlafmedizin |
| --- | --- |
| Principal investigator | Study nurse; Bettina Börner |

| Place/Date |  | Signature |
| --- | --- | --- |

| Principal investigator | Dr. med. M. Tini |
| --- | --- |

| Place/Date |  | Signature |
| --- | --- | --- |

| Principal investigator | Dr. med. P. Fachinger |
| --- | --- |

| Place/Date |  | Signature |
| --- | --- | --- |

Table of Contents

Study synopsis 9

study summary in local language 12

Abbreviations 14

Study schedule 15

1. STUDY ADMINISTRATIVE STRUCTURE 16

1.1 Sponsor, Sponsor-Investigator 16

1.2 Principal Investigator(s) 16

1.3 Statistician ("Biostatistician") 17

1.4 Laboratory 17

1.5 Monitoring institution 17

1.6 Data Safety Monitoring Committee 17

1.7 Any other relevant Committee, Person, Organisation, Institution 17

2. ETHICAL AND REGULATORY ASPECTS 19

2.1 Study registration 19

2.2 Categorisation of study 19

2.3 Competent Ethics Committee (CEC) 20

2.4 Competent Authorities (CA) 20

2.5 Ethical Conduct of the Study 20

2.6 Declaration of interest 20

2.7 Patient Information and Informed Consent 21

2.8 Participant privacy and confidentiality 21

2.9 Early termination of the study 21

2.10 Protocol amendments 22

3. Background and Rationale 23

3.1 Background and Rationale 23

3.2 Investigational Product (treatment, device) and Indication 23

3.3 Preclinical Evidence 23

3.4 Clinical Evidence to Date 23

3.5 Dose Rationale / Medical Device: Rationale for the intended purpose in study (pre-market MD) 24

3.6 Explanation for choice of comparator (or placebo) 24

3.7 Risks / Benefits 24

3.8 Justification of choice of study population 24

4. STUDY OBJECTIVES 26

4.1 Overall Objective 26

4.2 Primary Objective 26

4.3 Secondary Objectives 26

4.4 Safety Objectives 26

5. STUDY OUTCOMES 27

5.1 Primary Outcome 27

5.2 Secondary Outcomes 27

5.3 Other Outcomes of Interest 27

5.4 Safety Outcomes 27

6. STUDY DESIGN 28

6.1 General study design and justification of design 28

6.2 Methods of minimising bias 28

6.2.1 Randomisation 28

N.A. 28

6.2.2 Blinding procedures 28

6.2.3 Other methods of minimising bias 28

6.3 Unblinding Procedures (Code break) 29

7. STUDY POPULATION 30

7.1 Eligibility criteria 30

7.2 Recruitment and screening 31

7.3 Assignment to study groups 31

7.4 Criteria for withdrawal / discontinuation of participants 31

8. STUDY INTERVENTION 32

8.1 Identity of Investigational Products (treatment / medical device) 32

8.1.1 Experimental Intervention (treatment / medical device) 32

8.1.2 Control Intervention (standard/routine/comparator treatment / medical device) 32

8.1.3 Packaging, Labelling and Supply (re-supply) 32

8.1.4 Storage Conditions 32

8.2 Administration of experimental and control interventions 32

8.2.1 Experimental Intervention 32

8.2.2 Control Intervention 33

8.3 Dose / Device modifications 33

8.4 Compliance with study intervention 33

8.5 Data Collection and Follow-up for withdrawn participants 33

8.6 Trial specific preventive measures 33

8.7 Concomitant Interventions (treatments) 33

8.8 Study Drug / Medical Device Accountability 33

8.9 Return or Destruction of Study Drug / Medical Device 34

9. STUDY ASSESSMENTS 35

9.1 Study flow chart(s) / table of study procedures and assessments 35

9.2 Assessments of outcomes 35

9.2.1 Assessment of primary outcome 35

9.2.2 Assessment of secondary outcomes 35

9.2.3 Assessment of other outcomes of interest 35

9.2.4 Assessment of safety outcomes 35

9.2.5 Assessments in participants who prematurely stop the study 36

9.3 Procedures at each visit 36

9.3.1 Split into subtitles by type of visit 36

9.3.2 Split into subtitles by type of visit 36

9.3.3 Split into subtitles by type of visit 36

10. SAFETY 37

10.1 Drug studies 37

10.1.1 Definition and assessment of (serious) adverse events and other safety related events 37

10.1.2 Reporting of serious adverse events (SAE) and other safety related events 38

10.1.3 Follow up of (Serious) Adverse Events 39

10.2 Medical Device Category C studies 40

10.2.1 Definition and Assessment of (Serious) Adverse Events and other safety related events 40

10.2.2 Reporting of (Serious) Adverse Events and other safety related events 41

10.2.3 Follow up of (Serious) Adverse Events 41

10.3 Medical Device Category A studies 42

10.3.1 Definition and Assessment of safety related events 42

10.3.2 Reporting of Safety related events 42

11. STATISTICAL METHODS 43

11.1 Hypothesis 43

11.2 Determination of Sample Size 43

11.3 Statistical criteria of termination of trial 43

11.4 Planned Analyses 43

11.4.1 Datasets to be analysed, analysis populations 43

11.4.2 Primary Analysis 43

11.4.3 Secondary Analyses 43

11.4.4 Interim analyses 44

11.4.5 Safety analysis 44

11.4.6 Deviation(s) from the original statistical plan 44

11.5 Handling of missing data and drop-outs 44

12. QUALITY ASSURANCE AND CONTROL 45

12.1 Data handling and record keeping / archiving 45

12.1.1 Case Report Forms 45

12.1.2 Specification of source documents 45

12.1.3 Record keeping / archiving 45

12.2 Data management 45

12.2.1 Data Management System 46

12.2.2 Data security, access and back-up 46

12.2.3 Analysis and archiving 46

12.2.4 Electronic and central data validation 46

12.3 Monitoring 46

12.4 Audits and Inspections 46

12.5 Confidentiality, Data Protection 46

12.6 Storage of biological material and related health data 46

13. PUBLICATION AND DISSEMINATION POLICY 48

14. FUNDING AND SUPPORT 49

14.1 Funding 49

14.2 Other Support 49

15. INSURANCE 49

16. REFERENCES 50

17. APPENDICES 51

Study synopsis

(ClinO, Appendix 3, 1.1, 2.1, 3.1, 4.1; Appendix 5, 2b; AGEK Summary)

| Sponsor / Sponsor-Investigator | PD Dr.med.S.Irani, Kantonsspital Aarau |
| --- | --- |
| Study Title: | Change of olfactory performance after initiation of CPAP therapy in sleep apnea: a longitudinal study |
| Short Title / Study ID: | Olfactory performance after initiation of CPAP therapy |
| Protocol Version and Date: | Version 01; 08.08.2014 |
| Trial registration: | N.A. |
| Study category and Rationale | **Diese Studie ist ein Forschungsprojekt mit Personen, die mit einer Erhebung von gesundheitsbezogenen Personendaten verbunden sind. Es sind Routine-Sprechstunden-Patienten als Studienteilnehmer vorgesehen**. **Dies** **entspricht der Kategorie A.** |
| Clinical Phase: | N.A. |
| Background and Rationale: | **Die Therapie mit „continous positive airway pressure“ (CPAP), welche in der Regel via Nasenmaske appliziert wird, ist die Therapie der Wahl bei diagnostiziertem Schlaf-Apnoe-Syndrom. Wir beobachten im klinischen Alltag immer wieder Patienten, welche nach Beginn dieser Therapie über störende Sensationen im Bereich der Nasenschleimhaut klagen. Es existieren keine wissenschaftlichen Daten zur Frage, ob eine CPAP-Therapie den Geruchssinn beeinträchtigen kann. Dies ist insbesondere angesichts des bekannten Einflusses des Geruchsinnes auf die Lebensqualität eine relevante klinische Fragestellung.** |
| Objective(s): | Primär ist eine Geruchssinnmessung vor und 8-12 Wochen nach Beginn der CPAP Therapie mit einer standardisierten Testbatterie vorgesehen.  **Sekundär wird die Lebensqualität mit zwei standardisierten Fragebögen gemessen und die subjektive Geruchsempfindung im CRF abgefragt.** |
| Outcome(s): | Der primäre Endpunkt ist die Veränderung des Geruchssinnes nach 8-12 Wochen CPAP-Therapie. Sekundäre Endpunkte sind Änderung der Lebensqualität nach ebendieser Zeit, getestet anhand zweier standardisierten Fragebögen und einer subjektiven Geruchssineinschätzung. |
| Study design: | Es handelt sich um eine klinische Longitudinalstudie. |
| Inclusion / Exclusion criteria: | Eingeschlossen werden insgesamt 30 deutschsprachige Patienten unserer Schlafsprechstunde, welche zwischen 18 und 80 Jahre alt sind, sofern sie einverstanden sind und eine CPAP Therapie benötigen (AHI>10). Zur Ermittlung des AHI wird die Summe aus der Anzahl von Apnoen und Hypopnoen gebildet, mit 60 multipliziert und durch die Gesamtschlafzeit („Total sleep time“, einer Angabe in Minuten) dividiert. Dieser Wert wird via Polygraphie vor Beginn der Therapie ermittelt.  Ausgeschlossen werden Patienten, die nicht deutschsprechend sind und ebenfalls Patienten, die mit der Teilnahme nicht einverstanden sind oder bekannterweise unter Erkrankungen der Nasenhaupt und -nebenhöhle leiden. Auch stellt ein akuter Infekt der Atemwege ein Ausschlusskriterium dar.  **Ausschlusskriterien während der Studie sind: CPAP-Therapie <5h/d, Rauchstopp, neue Erkrankung der Nasenhaupt- und nebenhöhle** |
| Measurements and procedures: | Methode: Nicht invasiver, validierter Geruchssinnmessungstest mit dem Sniffin Sticks-Test (ergibt einen sogenannten TDI- Wert (Schwelle, Diskrimination, Identifikation) und 2 Fragebögen zur Lebensqualität (FLZ, HADS-D)  Sowohl die Untersuchung des Geruchssinnes, wie auch die Fragebogen werden vor dem Beginn der CPAP Therapie und nach 8-12 Wochen unter etablierter CPAP Therapie durchgeführt bzw. erhoben. Dies ergibt einen Mehrzeitaufwand für die eingeschlossenen Studienteilnehmer von ca. 30 Minuten pro Test, d.h. insgesamt ca. 1Stunde |
| Study Product / Intervention: | Es sind Routine-Sprechstunden-Patienten als Studienteilnehmer vorgesehen. Wir werden keine Kontrollgruppe verwenden, da es ethisch nicht vertretbar ist und es keine vernünftige andere Alternativtherapie gibt. |
| Control Intervention (if applicable): | N.A. |
| Number of Participants with Rationale: | In einer früheren Studie mit rund 90 Probanden haben wir einen TDI Mittelwert von 29 mit einer Standardabweichung von 6 gefunden. Wir nehmen entsprechend früheren Arbeiten an, dass eine Änderung des TDI um 4 Punkte als klinisch relevant angesehen werden kann. Nach Lehr kann bei einer Power von 80% und einem Signifikanzniveau von 0.05 eine Gruppengrösse von rund 30 errechnet werden. |
| Study Duration: | 12 Monate |
| Study Schedule: | November 2014 bis Oktober 2015 |
| Investigator(s): | Study nurse Bettina Börner  PD Dr.med. S. Irani  Abteilung für Pneumologie und Schlafmedizin  Kantonsspital Aarau  Tellstrasse  5001 Aarau |
| Study Centre(s): | Single-centre -study |
| Statistical Considerations: | Die statistische Auswertung erfolgt elektronisch mittels nicht-parametrischen statistischen Methoden (Wilcoxon-Test) |
| GCP Statement: | **This study will be conducted in compliance with the protocol, the current version of the Declaration of Helsinki, the ICH-GCP or ISO EN 14155 (as far as applicable) as well as all national legal and regulatory requirements**. |

study summary in local language

Die obstruktive Schlafapnoe ist eine sehr häufige Krankheit. Die meist via Nasenmaske durchgeführte CPAP Therapie ist die Therapie der Wahl.

Die klinische Beobachtung, dass Patienten unter CPAP Therapie vermehrt Irritationen im Bereich der Nasenschleimhaut verspüren, wird gestützt durch klinische Daten, dass eine CPAP Therapie mit einer Entzündung im Bereich der Nasenschleimhaut einhergehen kann. Ob dies einen Einfluss auf den Geruchssinn hat, ist unbekannt.

In einer früheren Studie konnten wir zeigen, dass ein verminderter Geruchssinn mit einer signifikant verminderten Lebensqualität in mehreren Domänen einhergeht. Zudem konnten wir nachweisen, dass das subjektive Empfinden hinsichtlich einer Funktionsstörung des eigenen Geruchssinnes sehr wenig sensitiv und spezifisch ist.

Wir möchten systematisch untersuchen, ob sich der Geruchssinn nach Beginn einer CPAP Therapie verschlechtert, ob sich ein Einfluss auf die Lebensqualität finden lässt und wie die Probanden ihren Geruchssinn selber einschätzen.

Für die Studienteilnehmenden bestehen keine Belastungen, auch hinsichtlich des zeitlichen Aufwandes kommt es nur zu einem sehr geringen Mehraufwand, da die Untersuchung des Geruchssinnes und das Ausfüllen des Fragebogen zur Lebensqualität während einer regulären Kontrolle erfolgen.

Methoden:

Der Sniffin Sticks Test 4-6 beinhaltet drei Module, in denen die Fähigkeit zur Identifikation und Diskriminierung von Gerüchen überprüft wird und einem Test, in dem die Riechschwelle für Butanol getestet wird. Mit dem Identifikationtstest kann grob orientierend die Frage beantwortet werden, ob der Patient ein normales oder vermindertes Riechvermögen besitzt. Es wird versucht, alltägliche Gerüche anhand einer Liste mit je vier Begriffen zu identifizieren. Es werden insgesamt 16 Gerüche angeboten. Der Diskrimierungstest basiert auf Unterscheidung von Gerüchen zwischen drei Duftdarbietungen. Dabei wird zweimal der gleiche Geruch angeboten und einmal ein andersriechender. Die Aufgabe der Teilnehmer ist, den jeweils anders riechenden Geruch anzugeben. Diese Vergleiche werden für 16 Duft-Tripplets angeboten. Die Riechschwelle für n-Butanol wird in einem sogenannten "Staircase" Verfahren ermittelt. Nach der Bestimmung einer Startkonzentration des Duftes wird die Verdünnung von Butanol festgestellt, bei der der Geruch gerade eben von nicht-riechenden Proben unterschieden werden kann. Um möglichst gute, aussagekräftige Ergebnisse zu erreichen, werden die Untersuchungen in einem ungestörten, gut belüfteten Raum durchgeführt. Während der Bestimmung der Riechschwelle und der Diskrimiation von Gerüchen tragen die Teilnehmer eine Augenbinde. Das Ergebnis der Tests wird als Summe der Ergebnisse der drei Untertest ermittelt, als sogenannter SDI- Wert. ( Schwelle, Diskrimination, Identifikation ). Dabei gilt ein Wert von mehr als 30 als normal, ein Wert von 30 und weniger bezeichnet eine Hyposmie und ein Wert von 15 und weniger spricht für eine funktionelle Anosmie im Sinne eines kompletten Riechverlustes, bzw. eines extrem abgeschwächten Riechvermögens. Mittels eines Fragebogen zum Erfassen der Lebensqualität, möchten wir feststellen, ob Patienten mit eingeschränkten Riechvermögen sich in Ihrer Lebensqualität beeinträchtig fühlen.

Bei Rauchern wird der Test frühestens zwei Stunden nach dem letzten Zigarettenkonsum durchgeführt.

Die verwendeten Standardfragebogen betreffen verschiedene Aspekte der Lebensqualität und sind in deutscher Sprache validiert.

Die ‚Fragen zur Lebenszusfriedenheit’ (FLZ) 7 bestehen aus 2x8 Modulen, wobei jedes Modul sowohl in Hinblick auf die subjektive Wertigkeit, wie auch in Hinblick auf die subjektive Zufriedenheit beantwortet wird. Die beiden Werte werden dann im Sinne einer ‚gewichteten Zufriedenheit’ (weighted satisfaction) mittels der Formel wS= importance rating x [(2 x satisfaction rating) – 3] kombiniert. Der Fragebogen besteht aus einem allgemeinen und einem Gesundheits-spezifischen Teil. Esbestehen ausgedehnte Vergleichsdaten bei gesunden und kranken Populationen

Die ‚Hospital anxiety depression scale’ (HADS-D) 8 misst Angst und Depressivität; sie wird vor allem bei körperlich kranken Patienten eingesetzt. Die HADS-D besteht aus sieben Items für die beiden Dimensionen Angst und Depressivität mit einer 4-Punkte-Likert-Skala. Der deutschsprachige Fragebogen wurde unter anderem mittels einer Normierungstudie validiert. Ein Gesamt wert von > 10 in jeder Skala gilt als als Hinweis auf eine kinisch relevante Angst- oder depressive Störung, ein Gesamtwert zwischen 8 und 10 liegt im Grenzbereich, ein Gesamtwert von < 8 wird als klinisch unauffällig interpretiert.

Sowohl die Untersuchung des Geruchssinnes, wie auch die Fragebogen werden unmittelbar vor dem Beginn der CPAP Therapie und nach 8-12 Wochen unter etablierter CPAP Therapie durchgeführt bzw. erhoben.

Abbreviations

| AE | Adverse Event |
| --- | --- |
| CA | Competent Authority (e.g. Swissmedic) |
| CEC | Competent Ethics Committee |
| CRF | Case Report Form |
| ClinO | Ordinance on Clinical Trials in Human Research *(in German: KlinV, in French: OClin)* |
| eCRF | Electronic Case Report Form |
| CTCAE | Common terminology criteria for adverse events |
| DSUR | Development safety update report |
| GCP | Good Clinical Practice |
| IB | Investigator’s Brochure |
| Ho | Null hypothesis |
| H1 | Alternative hypothesis |
| HFG | Humanforschungsgesetz (Law on human research) |
| HMG | Heilmittelgesetz |
| HRA | Federal Act on Research involving Human Beings |
| IMP | Investigational Medicinal Product |
| IIT | Investigator-initiated Trial |
| ISO | International Organisation for Standardisation |
| ITT | Intention to treat |
| KlinV | Verordnung über klinische Versuche in der Humanforschung *(in English: ClinO, in French OClin)* |
| LPTh | Loi sur les produits thérapeutiques |
| LRH | Loi fédérale relative à la recherche sur l’être humain |
| MD | Medical Device |
| OClin | Ordonnance sur les essais cliniques dans le cadre de la recherche sur l'être humain *(in German : KlinV, in English : ClinO)* |
| PI | Principal Investigator |
| SDV | Source Data Verification |
| SOP | Standard Operating Procedure |
| SPC | Summary of product characteristics |
| SUSAR | Suspected Unexpected Serious Adverse Reaction |
| TMF | Trial Master File |

Study schedule

(AGEK 4.2; SPIRIT #13; ICH E6 6.4.2)

| Study Periods | Screening | Follow-up 1 | Follow-up 2 |
| --- | --- | --- | --- |
| Visit | 1 | 1 | 1 |
| Time (hour, day, week) | 0 | 0-5d | 8-12week |
| Patient Information and Informed Consent | x |  |  |
| Demographics | x | x | x |
| Medical History | x | x | x |
| In- /Exclusion Criteria | x |  |  |
| Physical Examination | x |  |  |
| Other test | x | x | x |
| Primary Variables | x | x | x |
| Secondary Variables | x | x | x |

# STUDY ADMINISTRATIVE STRUCTURE

(ICH/E6 6.1.2-6.1.7; AGEK 1.1; SPIRIT 5a-d)

N. A.

## Sponsor, Sponsor-Investigator

(ICH/E6 6.1.2; AGEK 1.1; SPIRIT 5b)

ICH: Name and address of the sponsor …..

PD Dr.med. S. Irani

Abteilung für Pneumologie und Schlafmedizin

Kantonsspital Aarau

Tellstrasse

5001 Aarau

062/838 4470

Aufgabe: Patientenauswahl, Management, Analyse, Interpretation der Daten, Schreiben der Auswertung

## Principal Investigator(s)

(ICH/E6 6.1.5, 6.1.6; AGEK 1.1; SPIRIT 5a-d)

ICH: Name and title of the investigator(s) who is (are) responsible for conducting the trial, and the address and telephone number(s) of the trial site(s).

Bettina Börner, study nurse

Abteilung für Pneumologie und Schlafmedizin

Kantonsspital Aarau

Tellstrasse

5001 Aarau

062/838 4478

Dr.med. Mauro G. Tini

Abteilung für Pneumologie und Schlafmedizin

Kantonsspital Aarau

Tellstrasse

5001 Aarau

062/838 4479

Dr.med. Patrick Fachinger

Abteilung für Pneumologie und Schlafmedizin

Kantonsspital Aarau

Tellstrasse

5001 Aarau

062/838 5889

Aufgabe: Patientenauswahl, Management, Analyse, Interpretation der Daten, Schreiben der Auswertung

## Statistician ("Biostatistician")

(ICH/E6 6.1.7; SPIRIT 5a-d)

ICH: Name(s) and address(es) of the clinical laboratory(ies) and other medical and/or technical department(s) and/or institutions involved in the trial.

Das involvierte „research-Team“ (siehe auch 1.1, 1.2)

Abteilung für Pneumologie und Schlafmedizin

Kantonsspital Aarau

Tellstrasse

5001 Aarau

062/838 4470

## Laboratory

(ICH/E6 6.1.7; SPIRIT 5a-d)

ICH: Name(s) and address(es) of the clinical laboratory(ies) …………. involved in the trial.

N. A.

## Monitoring institution

(ICH/E6 6.1.2; SPIRIT 5a-d)

ICH: ICH: Name and address of the …. monitor (if other than the sponsor).

N. A.

## Data Safety Monitoring Committee

(ICH/E6 6.1.7; SPIRIT 5a-d)

ICH: Name(s) and address(es) of the clinical laboratory(ies) and other medical and/or technical department(s) and/or institutions involved in the trial.

Frau G. Zehnder

Abteilung für Pneumologie und Schlafmedizin

Kantonsspital Aarau

Tellstrasse

5001 Aarau

062/838 4470

## Any other relevant Committee, Person, Organisation, Institution

(ICH/E6 6.1.7; SPIRIT 5a-d)

ICH: Name(s) and address(es) of the clinical laboratory(ies) and other medical and/or technical department(s) and/or institutions involved in the trial.

N.A.

# ETHICAL AND REGULATORY ASPECTS

(ICH/E6 6.12; AGEK 11; SPIRIT #24, 5)

ICH: Description of ethical considerations relating to the trial.

The decision of the CEC and Swissmedic/foreign competent authority concerning the conduct of the study will be made in writing to the Sponsor-Investigator before commencement of this study. The clinical study can only begin once approval from all required authorities has been received. Any additional requirements imposed by the authorities shall be implemented.

## Study registration

(ClinO, Art. 1d, 64; SPIRIT #2a-b)

N.A.

## Categorisation of study

(ClinO, Art. 19, 20, App 3, 1.1)

Risk A, no intervention

## Competent Ethics Committee (CEC)

(ClinO, Art 24-29; SPIRIT #24)

Mention that the responsible investigator at Competent Ethics Committee (CEC) is sought for the clinical study.

Unanticipated problems will be reported to the CEC within 5 days.

## Competent Authorities (CA)

(ClinO, Art. 23, 27, 30-39, 42, 43, 46-48, 57; SPIRIT #24)

N. A.

## Ethical Conduct of the Study

(ClinO, Art. 5; AGEK 11; ICH E6 6.12, 6.2.5)

ICH: A statement that the trial will be conducted in compliance with the protocol, GCP and the applicable regulatory requirement(s).

The study will be carried out in accordance to the protocol and with principles enunciated in the current version of the Declaration of Helsinki, the guidelines of Good Clinical Practice (GCP) issued by ICH, in case of medical device: the European Directive on medical devices 93/42/EEC and the ISO Norm 14155 and ISO 14971, the Swiss Law and Swiss regulatory authority’s requirements. The CEC and regulatory authorities will receive annual safety and interim reports and be informed about study stop/end in agreement with local requirements.

## Declaration of interest

(ClinO, Art. 3b; SPIRIT #28)

No financial or other interests exist.

## Patient Information and Informed Consent

(ClinO, Art. 7-9, Art. 15-17, Appendix 3, 1.4, 2.4, 3.4, 4.3, Appendix 4, 3.6; AGEK submission checklist item 5; SPIRIT #26, 32)

The investigators will explain to each participant the nature of the study, its purpose, the procedures involved, the expected duration, the potential risks and benefits and any discomfort it may entail. Each participant will be informed that the participation in the study is voluntary and that he/she may withdraw from the study at any time and that withdrawal of consent will not affect his/her subsequent medical assistance and treatment.

The participant must be informed that his/her medical records may be examined by authorised individuals other than their treating physician.

All participants for the study will be provided a participant information sheet and a consent form describing the study and providing sufficient information for participant to make an informed decision about their participation in the study.

The patient information sheet and the consent form will be submitted to the CEC and to the competent authority (as applicable) to be reviewed and approved. The formal consent of a participant, using the approved consent form, must be obtained before the participant is submitted to any study procedure.

The participant should read and consider the statement before signing and dating the informed consent form, and should be given a copy of the signed document. The consent form must also be signed and dated by the investigator (or his designee) and it will be retained as part of the study records.

## Participant privacy and confidentiality

(ClinO, Art. 18; ICH/E6 6.10; AGEK 12.2, SPIRIT #27)

ICH:

The investigator affirms and upholds the principle of the participant's right to privacy and that they shall comply with applicable privacy laws. Especially, anonymity of the participants is guaranteed when presenting the data at scientific meetings or publishing them in scientific journals.

Individual subject medical information obtained as a result of this study is considered confidential and disclosure to third parties is prohibited. Subject confidentiality will be further ensured by utilising subject identification code numbers to correspond to treatment data in the computer files.

For data verification purposes, authorised representatives of the Sponsor (-Investigator), a competent authority (e.g. Swissmedic), or an ethics committee may require direct access to parts of the medical records relevant to the study, including participants’ medical history.

## Early termination of the study

(ClinO Art. 47; ICH/E6 6.4.6; SPIRIT #21b)

ICH: A description of the "stopping rules" or "discontinuation criteria" for individual participants, parts of trial and entire trial.

The Sponsor-Investigator may terminate the study prematurely according to certain circumstances, for example:

In case of the unlikely circumstance that the validate test should cause any harm.

## Protocol amendments

(ClinO, Art. 29, 34, 55; SPIRIT #25)

Substantial amendments are only implemented after approval of the CEC and CA respectively.

Under emergency circumstances, deviations from the protocol to protect the rights, safety and well-being of human subjects may proceed without prior approval of the sponsor and the CEC/CA. Such deviations shall be documented and reported to the sponsor and the CEC/CA as soon as possible.

All Non-substantial amendments are communicated to the CA as soon as possible if applicable and to the CEC within the Annual Safety Report (ASR).

# Background and Rationale

(ICH 6.2; AGEK 3; SPIRIT #6)

## Background and Rationale

(ICH/E6 6.2; AGEK 3.1; SPIRIT #6)

Die Therapie mit „continous positive airway pressure“ (CPAP), welche in der Regel via Nasenmaske appliziert wird, ist die Therapie der Wahl bei diagnostiziertem Schlaf-Apnoe-Syndrom. Wir beobachten im klinischen Alltag immer wieder Patienten, welche nach Beginn dieser Therapie über störende Sensationen im Bereich der Nasenschleimhaut klagen. Es existieren keine wissenschaftlichen Daten zur Frage, ob eine CPAP-Therapie den Geruchssinn beeinträchtigen kann. Dies ist insbesondere angesichts des bekannten Einflusses des Geruchsinnes auf die Lebensqualität eine relevante klinische Fragestellung.

## Investigational Product (treatment, device) and Indication

(ICH/E6 6.2.1; AGEK 2; SPIRIT #6)

ICH: Name and description of the investigational product(s).

N. A.

## Preclinical Evidence

(ICH/E6 6.2.2; SPIRIT #6a)

ICH: A summary of findings from nonclinical studies that potentially have clinical significance …..

N. A.

## Clinical Evidence to Date

(ICH/E6 6.2.2; SPIRIT #6a)

ICH: A summary of findings from … and from clinical trials that are relevant to the trial.

Schlafapnoe ist eine häufige Krankheit. In den meisten Fällen wird die Behandlung der CPAP-Therapie mit Hilfe einer Nasenmaske (nCPAP) durchgeführt

. Die klinische Beobachtung, dass nCPAP führt häufig zu Reizungen der Nasenschleimhaut durch wissenschaftliche Daten, die eine erhöhte entzündliche Aktivität der Nasenschleimhaut nach Beginn der nCPAP zeigen unterstützt. Ihre Auswirkungen auf die Riechleistung ist unbekannt.

In einer Längsschnittstudie wollen wir die Veränderungen des Riechleistung nach Beginn der nCPAP-Therapie in einer Gruppe von aufeinanderfolgenden ambulanten Patienten unserer Abteilung zu untersuchen. Wir vermuten, dass der Riechleistung kann nach Beginn der nCPAP-Therapie zu verringern. Darüber hinaus könnte dies Auswirkungen auf bestimmte Domänen der Lebensqualität.

Sleep apnea is a high incidence disorder. In most cases the treatment consists of CPAP therapy which is applied with the aid of a nasal mask (nCPAP). The clinical observation that nCPAP frequently results in irritation of the nasal mucosa is supported by scientific data that show increased inflammatory activity of the nasal mucosa after initiation of nCPAP ^[[1]](#footnote-1)^ ^[[2]](#footnote-2)^. Its impact on olfactory performance is unknown.

In a longitudinal study we aim to investigate changes of the olfactory performance after initiation of nCPAP therapy in a group of consecutive outpatients of our department. We hypothesize that the olfactory performance might decrease after initiation of nCPAP therapy. Furthermore, this could have consequences on certain domains of the quality of life.

## Dose Rationale / Medical Device: Rationale for the intended purpose in study (pre-market MD)

(ICH/E6 6.2.4; SPIRIT #6a)

ICH: Description of and justification for the route of administration, dosage, dosage regimen, and treatment period(s).

N. A.

## Explanation for choice of comparator (or placebo)

(AGEK 11.3; SPIRIT #6b)

N.A.

## Risks / Benefits

(ClinO, Appendix 4, 3.5; Art 25d2; ICH/E6 6.2.3; AGEK 11.1; SPIRIT #6a; MD: ISO 14155 Annex A & ISO 14971)

ICH: Summary of the known and potential risks and benefits, if any, to human subjects.

Da nichts spezielle getan wird, wird kein Risiko an der zu untersuchenden Population erwartet.

## Justification of choice of study population

(ClinO, Art 25d4, Art. 15-17; ICH/E6 6.2.6; AGEK 11.2)

ICH: Description of the population to be studied.

N. A.

# STUDY OBJECTIVES

(ICH/E6 6.3; AGEK 3; SPIRIT #7)

ICH: A detailed description of the objectives and the purpose of the trial.

## Overall Objective

Die Beeinflussung der CPAP-Therapie des Geruchssinnes und somit die Lebensqualität

## Primary Objective

Geruchssintest

## Secondary Objectives

Quality of life-Fragebogen

## Safety Objectives

N.A.

# STUDY OUTCOMES

(ICH/E6 6.4.1; AGEK 4.1; SPIRIT #12)

ICH: A specific statement of the primary endpoints and the secondary endpoints, if any, to be measured during the trial.

## Primary Outcome

Der primäre Endpunkt ist die Veränderung des Geruchssinnes nach 8-12 Wochen CPAP-Therapie mittels TDI-Wert.

## Secondary Outcomes

Sekundäre Endpunkte sind Änderungen der Lebensqualität nach ebendieser Zeit, getestet anhand zweier standardisierten Fragebögen und einer subjektiven Geruchssineinschätzung.

## Other Outcomes of Interest

Weiter ergeben sich Endpunkte durch anatomische Veränderungen des Geruchssinnes, wie zum Beispiel bei Erkältungen, Nasenoperationen, Unfälle, in welchem der Riechapparat involviert ist.

## Safety Outcomes

N.A.

# STUDY DESIGN

(ICH/E6 6.4; AGEK 4; SPIRIT #8)

## General study design and justification of design

(ICH/E6 6.4.2, 6.4.5; AGEK 4.2; SPIRIT #8)

ICH: The scientific integrity of the trial and the credibility of the data from the trial depend substantially on the trial design.

ICH: A description of the type/design of trial to be conducted (e.g., double-blind, placebo-controlled, parallel design) and a schematic diagram of trial design, procedures and stages.

ICH: The expected duration of subject participation, and a description of the sequence and duration of all trial periods, including follow-up, if any.

Es handelt sich um eine klinische Longitudinalstudie. Für die geplante Fragestellung bestes Studiendesign da interindividuelle Unterschiede des Riechvermögens relativ gross sind.

## Methods of minimising bias

(ICH/E6 6.4.3; AGEK 4.3; SPIRIT #16, 17)

ICH: A description of the measures taken to minimize/avoid bias, including: Randomization, Blinding.

### Randomisation

### N. A.

### Blinding procedures

N. A.

### Other methods of minimising bias

Verwendung von validierten Tests.

## Unblinding Procedures (Code break)

(ICH/E6 6.4.8; AGEK 4.2; SPIRIT #17b)

ICH: Maintenance of trial treatment randomization codes and procedures for breaking codes.

N. A.

# STUDY POPULATION

(ICH/E6 6.2.6, 6.4.6; AGEK 3.2, 5; SPIRIT #9, 10, 15, 16, 21)

ICH: Description of the population to be studied.

## Eligibility criteria

(ClinO, Art 25d5; ICH/E6 6.5.1&6.5.2; AGEK 5.2&5.3; SPIRIT #10)

ICH: Subject inclusion and exclusion criteria.

Eingeschlossen werden insgesamt 30 deutschsprachige Patienten unserer Schlafsprechstunde, welche zwischen 18 und 80 Jahre alt sind, sofern sie einverstanden sind und eine CPAP Therapie benötigen (AHI>10). Es handelt sich dabei um für die untersuchte Population representative Probanden.

Ausgeschlossen werden Patienten, die nicht deutschsprechend sind und ebenfalls Patienten, die mit der Teilnahme nicht einverstanden sind oder bekannterweise unter Erkrankungen der Nasenhaupt und -nebenhöhle leiden. Auch stellt ein akuter Infekt der Atemwege ein Ausschlusskriterium dar.

## Recruitment and screening

(ClinO, Art 25, Appendix 3, 1.4 & 1.6; AGEK 5.1; SPIRIT #15)

Im besagten Studienzeitfenster werden alle in Frage kommenden Patienten um eine Teilnahme erfragt.

## Assignment to study groups

(AGEK 5; SPIRIT #16)

N. A.

## Criteria for withdrawal / discontinuation of participants

(ClinO, Art 9; ICH/E6 6.5.3; SPIRIT #21b)

Subject withdrawal criteria (i.e., terminating investigational product treatment/trial treatment) and procedures specifying: a) When and how to withdraw subjects from the trial/ investigational product treatment. c) Whether and how subjects are to be replaced.

Wenn der Patient den Studienausschluss wünscht, wird er nicht in die Studie einbezogen.

# STUDY INTERVENTION

(SPIRIT #11)

## Identity of Investigational Products (treatment / medical device)

(ICH/E6 6.2.1, 6.4.2, 6.4.4; AGEK Checklist 2, item 3)

ICH: A description of the trial treatment(s) and the dosage and dosage regimen of the investigational product(s).

N. A.

### Experimental Intervention (treatment / medical device)

ICH: Name and description of the investigational product(s).

N. A.

### Control Intervention (standard/routine/comparator treatment / medical device)

ICH: Name and description of the investigational product(s).

N. A.

###

ICH: Also include a description of the dosage form, packaging, and labelling of the investigational product(s).

N. A.

### Storage Conditions

N. A.

## Administration of experimental and control interventions

(ICH/E6 6.4.4)

### Experimental Intervention

ICH: Description of and justification of the treatment(s) to be administered, including the name(s) of all the product(s), the dose(s), the dosing schedule(s), the route/mode(s) of administration, and the treatment period(s), including the follow-up period(s) for subjects for each investigational product treatment/trial treatment group/arm of the trial.

N. A.

### Control Intervention

ICH: Description of and justification of the treatment(s) to be administered, including the name(s) of all the product(s), the dose(s), the dosing schedule(s), the route/mode(s) of administration, and the treatment period(s), including the follow-up period(s) for subjects for each investigational product treatment/trial treatment group/arm of the trial.

N. A.

## Dose / Device modifications

(SPIRIT #11b)

N. A.

## Compliance with study intervention

(ICH/E6 6.6.3; AGEK Checklist 2, item 2; SPIRIT #11c)

ICH: Procedures for monitoring subject compliance.

N. A.

## Data Collection and Follow-up for withdrawn participants

(ICH/E6 6.5.3; AGEK 9.2; SPIRIT #18b)

ICH: ………..b) The type and timing of the data to be collected for withdrawn subjects. d) The follow-up for subjects withdrawn from investigational product treatment/trial treatment.

N. A.

## Trial specific preventive measures

(ICH/E6 6.6.2; AGEK 9; SPIRIT #11d)

ICH: Medication(s)/treatment(s) permitted (including rescue medication) and not permitted before and/or during the trial.

N. A.

## Concomitant Interventions (treatments)

(ICH/E6 6.6.2; AGEK 9; SPIRIT #11d)

ICH: Medication(s)/treatment(s) permitted (including rescue medication) and not permitted before and/or during the trial.

N. A.

## Study Drug / Medical Device Accountability

(ICH/E6 6.4.7; AGEK Checklist 2, item 1; SPIRIT 11c)

ICH: Accountability procedures for the investigational product(s), including the placebo(s) and comparator(s), if any.

N. A.

## Return or Destruction of Study Drug / Medical Device

(AGEK Checklist 2, item 1; SPIRIT 11c)

N. A.

# STUDY ASSESSMENTS

(ICH/E6 6.7, 6.8; AGEK 6, 7; SPIRIT #18a)

Describe procedures, measurements, collection, storage of samples taken, etc.

## Study flow chart(s) / table of study procedures and assessments

## 0-5d 8-12weeks

Screen Follow up 1 Follow up 2

(Assessment + therapy) (Assessment)

## Assessments of outcomes

ICH: Specification of the efficacy parameters. Specification of safety parameters.

### Assessment of primary outcome

ICH: Methods and timing for assessing, recording, and analysing of efficacy & safety parameters.

Primärer Endpunkt: Veränderung des Geruchssinn mittels validiertem Geruchsttest (TDI)

### Assessment of secondary outcomes

ICH: Methods and timing for assessing, recording, and analysing of efficacy & safety parameters.

Sekundärer Endpunkt: Veränderung der Lebensqualität mittels validiertem Lebensqualitäts-Fragebogen.

### Assessment of other outcomes of interest

ICH: Methods and timing for assessing, recording, and analysing of efficacy & safety parameters.

N. A.

### Assessment of safety outcomes

ICH E6 6.8: Specification of safety parameters. The methods and timing for assessing, recording, and analysing safety parameters

N. A.

#### Adverse events

keine

#### Laboratory parameters

Keine

#### Vital signs

Diese werden in der Screening-Visit erhoben

### Assessments in participants who prematurely stop the study

Es ist kein Follow up für diese Patienten realistisch. Dieses Vorgehen ist vertretbar, da keine Intervention durchgeführt wurde

## Procedures at each visit

Provide a verbal description of procedures at each visit according to study phase: e.g. screening, baseline, visits during intervention, close-out visit, follow-up visits. Includes additional tasks as scheduling of next visit, distribution of study medication, what is not a measurement to be described (bulleted list).

### Split into subtitles by type of visit

Screening: Standardmässige schlafmedizinische Konsultation inklusiv Polygraphie; zusätzlich Fragestellung nach der Einverständniserklärung

### Split into subtitles by type of visit

Follow up 1: Etablierung der CPAP-Therapie; Quality of life (QoL)-Fragebogen; Geruchssinnmessung (TDI); CRF-Bogen

### Split into subtitles by type of visit

Follow up 2: standardisierte CPAP-Geräte-Daten-Download; QoL-Fragebogen; Geruchssinnmessung (TDI); CRF-Bogen

# SAFETY

(ClinO Art. 37-43; ICH/E6 6.8; ISO14155 8.2.5, A.14; AGEK 4.1; SPIRIT # 22, 30)

Es sind keine sicherheitsrelevante Aspekte zu erwarten.

## Drug studies

The Sponsor’s SOPs provide more detail on safety reporting.

During the entire duration of the study, all adverse events (AE) and all serious adverse events (SAEs) are collected, fully investigated and documented in source documents and case report forms (CRF). Study duration encompassed the time from when the participant signs the informed consent until the last protocol-specific procedure has been completed, including a safety follow-up period.

### Definition and assessment of (serious) adverse events and other safety related events

ICH: Procedures for eliciting reports of and for recording … adverse event and intercurrent illnesses.

An **Adverse Event (AE)** is any untoward medical occurrence in a patient or a clinical investigation participant administered a pharmaceutical product and which does not necessarily have a causal relationship with the study procedure. An AE can therefore be any unfavourable and unintended sign (including an abnormal laboratory finding), symptom, or disease temporally associated with the use of a medicinal (investigational) product, whether or not related to the medicinal (investigational) product. [ICH E6 1.2]

A **Serious Adverse Event (SAE)** is classified as any untoward medical occurrence that:

- results in death,
- is life-threatening,
- requires in-patient hospitalization or prolongation of existing hospitalisation,
- results in persistent or significant disability/incapacity, or
- is a congenital anomaly/birth defect.

In addition, important medical events that may not be immediately life-threatening or result in death, or require hospitalisation, but may jeopardise the patient or may require intervention to prevent one of the other outcomes listed above should also usually be considered serious. [ICH E2A]

SAEs should be followed until resolution or stabilisation. Participants with ongoing SAEs at study termination (including safety visit) will be further followed up until recovery or until stabilisation of the disease after termination.

Assessment of Causality

Both Investigator and Sponsor-investigator make a causality assessment of the event to the study drug, based on the criteria listed in the ICH E2A guidelines:

| Relationship | Description |
| --- | --- |
| Definitely | Temporal relationship  Improvement after dechallenge*  Recurrence after rechallenge  (or other proof of drug cause) |
| Probably | Temporal relationship  Improvement after dechallenge  No other cause evident |
| Possibly | Temporal relationship  Other cause possible |
| Unlikely | Any assessable reaction that does not fulfil the above conditions |
| Not related | Causal relationship can be ruled out |
| *Improvement after dechallenge only taken into consideration, if applicable to reaction | |

Unexpected Adverse Drug Reaction

An “unexpected” adverse drug reaction is an adverse reaction, the nature or severity of which is not consistent with the applicable product information (e.g. Investigator’s Brochure for drugs that are not yet approved and Product Information for approved drugs, respectively). [ICH E2A]

Suspected Unexpected Serious Adverse Reactions (SUSARs)

The Sponsor-Investigator evaluates any SAE that has been reported regarding seriousness, causality and expectedness. If the event is related to the investigational product and is both serious and unexpected, it is classified as a SUSAR.

Assessment of Severity

### Reporting of serious adverse events (SAE) and other safety related events

(ClinO Art. 37)

ICH: Procedures for … reporting adverse event and intercurrent illnesses.

Reporting of SAEs

All SAEs must be reported immediately and within a maximum of 24 hours to the Sponsor-Investigator of the study. The Sponsor-Investigator will re-evaluate the SAE and return the form to the site.

SAEs resulting in death are reported to the local Ethics Committee (via local Investigator) within 7 days.

The other in the trial involved Ethics Committees receive SAEs resulting in death in Switzerland via Sponsor-Investigator within 7 days.

Reporting of SUSARs

A SUSAR needs to be reported to the local Ethics Committee (local event via local Investigator) and to Swissmedic for category B and C studies (via Sponsor-Investigator) within 7 days, if the event is fatal, or within 15 days (all other events).

The Sponsor-Investigator must inform all Investigators participating in the clinical study of the occurrence of a SUSAR. All in the trial involved Ethics Committees will be informed about SUSARs in Switzerland via Sponsor-Investigator according to the same timelines.

Reporting of Safety Signals

All suspected new risks and relevant new aspects of known adverse reactions that require safety-related measures, i.e. so called safety signals, must be reported to the Sponsor-Investigator within 24 hours. The Sponsor-Investigator must report the safety signals within 7 days to the local Ethics Committee (local event via local Investigator) and to Swissmedic in case of a category B or C study.

The Sponsor-Investigator must immediately inform all participating Investigators about all safety signals. The other in the trial involved Ethics Committees will be informed about safety signals in Switzerland via the Sponsor-Investigator.

Reporting and Handling of Pregnancies

Pregnant participants must immediately be withdrawn from the clinical study. Any pregnancy during the treatment phase of the study and within 30 days after discontinuation of study medication will be reported to the Sponsor-Investigator within 24 hours. The course and outcome of the pregnancy should be followed up carefully, and any abnormal outcome regarding the mother or the child should be documented and reported.

Periodic reporting of safety

An annual safety report is submitted once a year to the local Ethics Committee via local Investigator and to Swissmedic in case of a category B or C study via Sponsor-Investigator.

### Follow up of (Serious) Adverse Events

(ICH/E6 6.8.4; SPIRIT #30)

ICH: The type and duration of the follow-up of subjects after adverse events.

## Medical Device Category C studies

All **adverse events (AE)** including all **serious adverse events (SAE)** are collected, fully investigated and documented in the source document and appropriate case report form (CRF) during the entire study period, i.e. from patient’s informed consent until the last protocol-specific procedure, including a safety follow-up period. Documentation includes dates of event, treatment, resolution, assessment of seriousness and causal relationship to device and/or study procedure.

N. A.

### Definition and Assessment of (Serious) Adverse Events and other safety related events

(MD: ISO 14155)

Adverse Event (AE)

Any untoward medical occurrence, unintended disease or injury or any untoward clinical signs (including an abnormal laboratory finding) in participants, users or other persons whether or not related to the investigational medical device [ISO 14155: 3.2].

Adverse Device Effect (ADE)

Adverse event related to the use of an investigational medical device [ISO 14155: 3.1].

Serious Adverse Event (SAE)

Adverse event that:

- results in death, or
- led to a serious deterioration in health that either:
  - results in a life-threatening illness or injury, or
  - results in a permanent impairment of a body structure or a body function, or
  - required in-patient or prolonged hospitalisation, or
  - results in medical or surgical intervention to prevent life threatening illness, or
- led to fetal distress, death or a congenital abnormality or birth defect. [ISO 14155: 3.37].

Device deficiency

Inadequacy of a medical device related to its identity, quality, durability, reliability, safety or performance, such as malfunction, misuse or use error and inadequate labelling [ISO 14155: 3.15].

Health hazards that require measures

Findings in the trial that may affect the safety of study participants and, which require preventive or corrective measures intended to protect the health and safety of study participants.

Causal Relationship of Adverse Events

A causal relationship towards the medical device or study procedure should be rated as follows:

- **Not related:** The event is definitely not associated with device application or with study procedures; a relationship can be ruled out.
- **Possibly related:** The relationship between device application or study procedures and the event is possible, but other causes cannot definitely be ruled out.
- **Related:** The event is definitely associated with device application or study procedures.

Device deficiencies that might have led to an SAE are always related to the medical device.

### Reporting of (Serious) Adverse Events and other safety related events

Reporting to Sponsor-Investigator:

The following events are to be reported to the Sponsor-Investigator within 24 hours upon becoming aware of the event:

- All SAEs
- Health hazards that require measures
- Device deficiencies

The Sponsor-Investigator will evaluate SAEs with regard to causality and seriousness. Device deficiencies are assessed regarding their potential to lead to an SAE.

Pregnancies N.A.

Reporting to Authorities:

In Category C studies it is the local Investigator’s responsibility to report **serious adverse events** in Switzerland which are

- related or possibly related to the medical device under investigation
- related or possibly related to study procedures

within 7 days to the local Ethics Committee. The Sponsor-Investigator reports within the same timeline to Swissmedic (incl. events from abroad).

- **Health hazards** that require measures are reported within 2 days

All in the trial involved other Ethical Committees receive all mentioned reportable SAEs and health hazards having occurred in Switzerland via the Sponsor-Investigator within the same timeline. All participating investigators are informed regarding the occurrence of a health hazard.

Periodic safety reporting

In Category C studies a yearly safety update-report is submitted by the Investigator to the Ethics Committee and by the Sponsor-Investigator to Swissmedic.

### Follow up of (Serious) Adverse Events

(SPIRIT #30)

## Medical Device Category A studies

### Definition and Assessment of safety related events

Health hazards that require measures

N. A.

### Reporting of Safety related events

Reporting to Sponsor-Investigator:

Health hazard that require measures are reported to the Sponsor-Investigator within 24 hours upon becoming aware of the event:

Pregnancies

Reporting to Authorities:

In Category A studies it is the Investigator’s responsibility to report to the local Ethics Committee

- **Health hazards** that require measures within 2 days

All other in the trial involved Ethics Committees receive health hazards having occurred in Switzerland via the Sponsor-Investigator within the same timeline. All participating investigators are informed regarding the occurrence of a health hazard.

The Sponsor-Investigator will notify Swissmedic of reportable events, based on the national regulations for materiovigilance procedures.

# STATISTICAL METHODS

(ICH/E6 6.9; AGEK 8; SPIRIT # 14, 20)

Statistical considerations

ICH: A description of the statistical methods to be employed, including timing of any planned interim analysis(ses).

## Hypothesis

Hypothese: gemäss Powerkalkulation mittels nichtparametrischen Tests eine Verschlechterung des Geruchssinnes detektiert wurden

## Determination of Sample Size

ICH: The number of subjects planned to be enrolled. In multicentre trials, the numbers of enrolled subjects projected for each trial site should be specified. Reason for choice of sample size, including reflections on (or calculations of) the power of the trial and clinical justification.

In einer früheren Studie mit rund 90 Probanden haben wir einen TDI Mittelwert von 29 mit einer Standardabweichung von 6 gefunden. Wir nehmen entsprechend früheren Arbeiten an, dass eine Änderung des TDI um 4 Punkte als klinisch relevant angesehen werden kann 9. Nach Lehr 10kann bei einer Power von 80% und einem Signifikanzniveau von 0.05 eine Gruppengrösse von rund 30 errechnet werden.

## Statistical criteria of termination of trial

ICH: A description of the "stopping rules" or "discontinuation criteria" for individual participants, parts of trial and entire trial.

N. A.

## Planned Analyses

ICH: A description of the statistical methods to be employed, including timing of any planned interim analysis(ses).

Der Vergleich vor und nach Beginn der CPAP Therapie erfolgt mittels nicht-parametrischen statistischen Methoden (Wilcoxon-Test).

### Datasets to be analysed, analysis populations

ICH: The selection of subjects to be included in the analyses (e.g., all randomized subjects, all dosed subjects, all eligible subjects, evaluable subjects).

### Primary Analysis

Nach Beenden der letzten Studienvisite des letzten Studienpatienten wird eine Statistik, wie in Punkt 11.4. erwähnt durch Sponsor durchgeführt.

### Secondary Analyses

Nach Beenden der letzten Studienvisite des letzten Studienpatienten wird eine Statistik, wie in Punkt 11.4. erwähnt durch Sponsor durchgeführt.

### Interim analyses

ICH 6.9.1: ...... including timing of any planned interim analysis(ses).

N. A.

### Safety analysis

N. A.

### Deviation(s) from the original statistical plan

(ICH/E6 6.9.6)

ICH: Procedures for reporting any deviation(s) from the original statistical plan (any deviation(s) from the original statistical plan should be described and justified in protocol and/or in the final report, as appropriate).

N. A.

## Handling of missing data and drop-outs

(ICH/E6 6.9.5; AGEK 8.5; SPIRIT 20c)

ICH: Procedure for accounting for missing, unused, and spurious data.

Entsprechend einer PP-Analyse.

# QUALITY ASSURANCE AND CONTROL

(ICH/E6 6.11, 6.13; AGEK 12; SPIRIT #19, 23, 27)

ICH: Quality Control and Quality Assurance Procedures

Qualitätskontrolle entsprechend einer klinischen Routine bezüglich Schlafmedizinischer Intervention (CPAP-Therapie)

## Data handling and record keeping / archiving

(ClinO, Art. 18, 45, 57, 62; ICH/E6 6.13; AGEK 12; SPIRIT #19, 27)

ICH: Data Handling and Record Keeping

Ausfüllen des CRF-Protokolls (siehe Anhang)

### Case Report Forms

(ICH/E6 6.4.9)

ICH: The identification of any data to be recorded directly on the CRFs (i.e., no prior written or electronic record of data), and to be considered to be source data.

Das CRF-Protokoll wird in Papier durch die Study nurse ausgefüllt.

### Specification of source documents

(ICH/E6 6.4.9)

ICH: The identification of any data to be recorded directly on the CRFs (i.e., no prior written or electronic record of data), and to be considered to be source data.

Bezugsquelle: CRF-Protokoll, CPAP-Therapie-Verlaufsprotokoll, Polygraphiprotokoll

### Record keeping / archiving

(ICH/E6 6.13)

ICH: Data Handling and Record Keeping

Alle Studiendaten werden minimum 10 Jahre aufbewahrt und archiviert.

## Data management

(ICH/E2; AGEK 12.2; SPIRIT #19)

Codierung: Patient wird ananymisiert und codiert

Die Daten in Papierformat werden in einem abschliessbaren Schrank der Study nurse gelagert und die ananymisierte Codierung sowie die Einverständniserklärung in einem separaten Schrank gelagert.

### Data Management System

Die Daten werden durch die study nurse in einem CRF in Papierformat gesichert und gelagert.

### Data security, access and back-up

Die Daten werden nur durch die Studynurse betreut und bearbeitet.

### Analysis and archiving

Die Datenanalyse erfolgt mittel einem elektronischen Statistikprogramm. Die Daten werden auf einem persönlichen Laptop gespeichert.

### Electronic and central data validation

N.A.

## Monitoring

(AGEK 12.1; SPIRIT #23)

Die Daten werden durch eine gezielte Visite des Monitors kontrolliert.

## Audits and Inspections

(ClinO, Art. 58, 59; AGEK 12.1; SPIRIT #23)

N. A.

## Confidentiality, Data Protection

(ClinO, Art. 18, 58; SPIRIT #27, 29)

Durchgeführt durch Study nurse, Sponsor, Monitor

## Storage of biological material and related health data

(ClinO, Art. 18; HVF Art. 28-32; SPIRIT #33)

N. A.

# PUBLICATION AND DISSEMINATION POLICY

(ICH/E6 6.15)

ICH: Publication policy, if not addressed in a separate agreement.

Die Resultate werden am europäischen Lungenforum (ERS) präsentiert und in einer Fachzeitschrift publiziert. Das Manuskript wird von dem Sponsor enworfen.

# FUNDING AND SUPPORT

(ClinO, Art. 25i; ICH/E6 6.14; SPIRIT #4)

Die Geruchssinnstudie wird von dem Forschungsrat des Kantonsspital Aarau unterstützt.

## Funding

(ClinO, Art. 25i)

ICH: Financing and insurance if not addressed in a separate agreement.

N. A.

## Other Support

(ClinO, Art. 25i)

ICH: Financing and insurance if not addressed in a separate agreement.

Es gibt keinen weiteren Support

# INSURANCE

(ClinO Art 12, 13; ICH/E6 6.14, AGEK 10.3; SPIRIT #30)

ICH: ….and insurance if not addressed in a separate agreement.

Eine Versicherung ist bei diesem Forschungsprojekt nicht nötig.

# REFERENCES

(ICH/E6 6.2.7)

ICH: References to literature and data that are relevant to the trial, and that provide background for the trial.

Provide a list of the references cited in the protocol.

1. Declaration of Helsinki, Version October 2013, (http://www.wma.net/en/30publications/10policies/b3/index.html )
2. International Conference on Harmonization (ICH, 1996) E6 Guideline for Good Clinical Practice. (http://www.ich.org/fileadmin/Public_Web_Site/ICH_Products/Guidelines/Efficacy/E6_R1/Step4/E6_R1__Guideline.pdf )
3. International Conference on Harmonization (ICH, 1997) E8 Guideline: General Considerations for Clinical Trials <http://www.ich.org/fileadmin/Public_Web_Site/ICH_Products/Guidelines/Efficacy/E8/Step4/E8_Guideline.pdf>)
4. Humanforschungsgesetz, HFG Bundesgesetz über die Forschung am Menschen (Bundesgesetz über die Forschung am Menschen, HFG) vom 30. September 2011/ Loi fédérale relative à la recherche sur l’être humain (loi relative à la recherche sur l’être humain, LRH) du 30 septembre 2011. (<http://www.bag.admin.ch/themen/medizin/00701/00702/07558/index.html?lang=de>)
5. Verordnung über klinische Versuche in der Humanforschung (Verordnung über klinische Versuche, KlinV) vom 20. September 2013 / Ordonnance sur les essais cliniques dans le cadre de la recherche sur l’être humain (Ordonnance sur les essais cliniques, OClin) du 20 septembre 2013. (http://www.bag.admin.ch/themen/medizin/00701/00702/12310/index.html?lang=de)
6. Heilmittelgesetz, HMG Bundesgesetz über Arzneimittel und Medizinprodukte (Heilmittelgesetz, HMG) vom 15. Dezember 2000/Loi fédérale sur les médicaments et les dispositifs médicaux (Loi sur les produits thérapeutiques, LPT) du 15 décembre 2000. (<http://www.admin.ch/ch/d/sr/8/812.21.de.pdf>)
7. ISO 14155:2011 Clinical investigation of medical devices for human subjects -- Good clinical practice (www.iso.org)
8. ISO 10993 Biological evaluation of medical devices (www.iso.org)
9. WHO, International Clinical Trials Registry Platform (ICTRP) (http://www.who.int/ictrp/en/)
10. Almendros I, Acerbi I, Vilaseca I et al. Continuous positive airway pressure (CPAP) induces early nasal inflammation. Sleep 2008; 31(1):127-131.
11. Shadan FF, Dawson A, Kline LE. CPAP therapy may evoke a local nasal inflammation in patients. Rhinology 2008; 46(4):347.
12. Irani S, Thomasius M, Schmid-Mahler C et al. Olfactory performance before and after lung transplantation: quantitative assessment and impact on quality of life. J Heart Lung Transplant 2010; 29(3):265-272.
13. Hummel T, Sekinger B, Wolf SR et al. 'Sniffin' sticks': olfactory performance assessed by the combined testing of odor identification, odor discrimination and olfactory threshold. Chem Senses 1997; 22(1):39-52.
14. Wolfensberger M, Schnieper I, Welge-Lussen A. Sniffin'Sticks: a new olfactory test battery. Acta Otolaryngol 2000; 120(2):303-306.
15. Wolfensberger M, Schnieper I. [Sniffin'Sticks: a new system for olfactory assessment in routine clinical practice]. HNO 1999; 47(7):629-636.
16. Henrich G, Herschbach P. Questions on life satisfaction (FLZ) - a short questionnaire for assessing subjective quality of life. Europ J Psychol Assess 2000; 16(3):150-159.
17. Zigmond AS, Snaith RP. The Hospital Anxiety and Depression Scale. Acta Psychiatrica Scandinavica 1983; 67(6):361-370.
18. Geissler K, Reimann H, Gudziol H et al. Olfactory training for patients with olfactory loss after upper respiratory tract infections. Eur Arch Otorhinolaryngol 2014; 271(6):1557-1562.
19. Lehr R. Sixteen S-squared over D-squared: a relation for crude sample size estimates. Stat Med 1992; 11(8):1099-1102.

# APPENDICES

ICH: (NOTE: Since the protocol and the clinical trial/study report are closely related, further relevant information can be found in the ICH Guideline for Structure and Content of Clinical Study Reports.)

1. Case Report Form (CRF)
2. Einverständniserklärung
3. Fragebogen HADS und Mini-QoL
4. Sniffin‘-Stick-Riechtest

1. [↑](#footnote-ref-1)
2. [↑](#footnote-ref-2)
