# Supplementary material for: Significant improvement of olfactory performance in sleep apnea patients after three months of nasal CPAP therapy – Observational study and randomized trial
Source: PLoS One. 2017 Feb 3;12(2):e0171087. doi: 10.1371/journal.pone.0171087 (PMC5291379; doi:10.1371/journal.pone.0171087)
Supplement: S5 File — Details of Study approval. (PDF) [file pone.0171087.s005.pdf]

Ethikkommission Nordwest- und Zentralschweiz EKNZ

Präsident
Prof. André P. Perruchoud
Vizepräsidenten
Prof. Gregor Schubiger
Dr. Marco Schärer

Herr
PD Dr. med. S. Irani
Pneumologie und Schlafmedizin
Kantonsspital Aarau
Tellstrasse
5001 Aarau

Basel, 18. November 2014

EKNZ 2014-335:
Change of olfactory performance after initiation of CPAP therapy in sleep apnea: a longitudinal study

Sehr geehrter Herr Dr. Irani

Besten Dank für Ihr Schreiben datiert vom 31. Oktober 2014 samt Beilagen. Die Ethikkommission Nordwest- und Zentralschweiz hat die nachfolgend erwähnten Dokumente (gemäss beiliegender Checkliste vom 31. Oktober 2014) zu oben genannter Studie zustimmend zur Kenntnis genommen und genehmigt:

- Forschungsplan - Version 02 vom 29. Oktober 2014
- Aufklärungsbogen / Information und Einwilligungserklärung - Version 02 vom 29. Oktober 2014
- Mitarbeiterliste - Version 01 vom 29. Oktober 2014 (bitte Versions-Nr. aktualisieren)
- CRF - Version 2 vom 29. Oktober 2014.

→ Die Auflagen der EKNZ (entsprechend der Verfügung vom 21. Oktober 2014) wurden somit erfüllt.

Ich hoffe, Ihnen mit dieser Bestätigung zu dienen und wünsche Ihnen für die Durchführung der Studie viel Erfolg.

Mit freundlichen Grüssen

Prof. A. P. Perruchoud
Präsident der Ethikkommission
Nordwest- und Zentralschweiz / EKNZ

Checkliste ab Januar 2014 HFV Anhang 2 Punkt 1 bis 3

Gesuchsunterlagen für Forschungsprojekte mit Personen, die mit der Entnahme von biologischem Material oder der Erhebung von gesundheitsbezogenen Personendaten verbunden sind

Vorlagen für die Dokumente sind auf www.swissethics.ch abrufbar.

EKNZ 2014-335 /31. Okt. 2014

Nr.	Dokumentbezeichnung	Datum/ Versions-nummer	Allfälliger Verweis auf anderes Dokument	KEK: Bemerkung (freilassen)
0	**Begleitschreiben**<br>– Rechnungsadresse muss vermerkt sein<br>– muss vom Gesuchsteller (Projektleitung oder Sponsor) signiert sein			
1a	**Basisformular, einschliesslich der Laien-Zusammenfassung des Forschungsplans für die Patienten in der(n) jeweiligen Landessprache(n) am Durchführungsort**<br>– muss von Projektleitung und falls zutreffend vom Sponsor signiert sein			
1b	**Zusammenfassung des Forschungsplans (Synopsis)für KEK-Mitglieder**<br>in der Landessprache der prüfenden KEK			
2	**Forschungsplan**<br>– muss von Projektleitung und falls zutreffend vom Sponsor signiert sein	29.10.2014<br>Version_02		
3a	**Aufklärungsbogen/Information und Einwilligungserklärung**<br>– in der(den) jeweiligen Landessprache(n) am jeweiligen Durchführungsort, den hier verantwortlichen Personen und Kontakten<br>– falls zutreffend auch Information für urteilsunfähige (z.B. Notfallpatienten, Demente), unmündige Personen, vertretungsberechtigte Personen (z.B. Eltern), oder für die schwangere PartnerIn des Studienteilnehmers<br>– für Sub-Studien separate Information (z.B. Zusatz-MRI-Untersuchung, pharmakokinetische Untersuchung)<br>– Information für die Weiterverwendung von Daten und Proben für zukünftige Forschungszwecke)	29.10.2014<br>Version_02		
3b	**Unterlagen betreffend Rekrutierung**<br>– namentlich Anzeige, Inseratetexte oder Rekrutierungsschreiben an den Patienten oder Hausarzt			

Nr.	Dokumentbezeichnung	Datum/ Versions-nummer	Allfälliger Verweis auf anderes Dokument	KEK: Bemerkung (freilassen)
4	**Weitere Unterlagen, die der teilnehmenden Person abgegeben werden**<br>– Patientenausweis, Tagebücher, Fragebogen in der jeweiligen Landessprache,<br>**oder andere Unterlagen, die im Rahmen der Studie verwendet werden**<br>– z.B. Interviewleitfaden, Scores, Fragebogen		CRF_29.10.2014_ Version_02	
5	**Angaben über Art und Ausmass/Wert der Entschädigung der teilnehmenden Personen**			
6	**Bei Forschungsprojekten der Kategorie B:**<br>– Versicherungsnachweis; oder<br>– anderer Nachweis der Sicherstellung für allfällige Schäden			
7	**Nachweis über sicheren Umgang mit biologischem Material und Personendaten**<br>– namentlich dessen, beziehungsweise deren Aufbewahrung			
8a	**Lebenslauf der Projektleitung und Nachweis der fachlichen Qualifikation (gemäss Art. 4 HFV)**<br>– signiert und datiert			
8b	**Auflistung der am Forschungsprojekt beteiligten Personen**<br>– einschliesslich ihrer Funktion und der entsprechenden fachlichen Kenntnisse	29.10.2014 Version_02		
9	**Nachweis über die Eignung und Verfügbarkeit der Infrastruktur am Durchführungsort**<br>– z.B.: Anzahl gleichzeitig durchgeführter Studien, Anzahl konkurrierender Studien, Vertretbarkeit der Geräteauslastung für das Forschungsprojekt etc.			
10	**Vereinbarung zwischen der Projektleitung und dem Sponsor oder weiteren Dritten**<br>– namentlich bezüglich der Finanzierung des Forschungsprojektes, der Zuteilung von Aufgaben, der Entschädigung der Projektleitung sowie bezüglich der Publikation<br>– muss von allen Parteien signiert sein			

Zusätzliche Gesuchsunterlagen für Forschungsprojekte, welche Untersuchungen mit Strahlenquellen umfassen (z.B. studienbegleitende Untersuchungen mit Röntgen, CT, Radiopharmazeutika für PET-Untersuchungen)

Einzureichen an die Ethikkommission:

Nr.	Dokumentbezeichnung	Datum/ Versionsnummer	Allfälliger Verweis auf anderes Dokument	KEK: Bemerkung (freilassen)
1	**Angaben zu wesentlichen Strahlenschutzaspekten, insbesondere eine Berechnung beziehungsweise Abschätzung der effektiven Strahlendosis, der Organdosis und allfälliger Tumordosen**			
2	**Die erforderliche Bewilligung für den Umgang mit Strahlenquellen oder radioaktiven Stoffen gemäss Artikel 28 des Strahlenschutzgesetzes vom 22. März 1991**[1]<br>Die einzuhaltenden Dosisgrenzwerte richten sich nach Art. 28 Abs. 3-5 nach der Strahlenschutzverordnung vom 22. Juni 1994[2]			

Zusätzliche Gesuchsunterlagen für Forschungsprojekte, welche Untersuchungen mit offenen oder geschlossenen radioaktiven Strahlenquellen umfassen und eine Stellungnahme des BAG nach Artikel 19 Absatz 2 erfordern

(Gilt ab einer Dosis von ≥ 5 mSV pro Person und Jahr beim Einsatz i) von in der Schweiz nicht zugelassenen Radiopharmazeutika. ii) von Radiopharmazeutika, welche zwar zugelassen sind, aber ausserhalb einer nuklearmedizinischen Routineuntersuchung verwendet werden iii)oder von anderen offenen oder geschlossenen radioaktiven Strahlenquellen. In allen anderen Fällen insbesondere bei Röntgenuntersuchungen oder CT ist die Stellungnahme des BAG nicht erforderlich).

Unter oben genannten Voraussetzungen zusätzlich ans BAG einzureichen:
(Der zuständigen Ethikkommission ist gleichzeitig mitzuteilen, dass diese Einreichung erfolgt ist)

Nr.	Dokumentbezeichnung	Datum/ Versionsnummer	Allfälliger Verweis auf anderes Dokument	KEK: Bemerkung (freilassen)
1	**Angaben zu den Eigenschaften des Radiopharmazeutikums, namentlich zur Pharmakokinetik, Qualität, Stabilität, radiochemische Reinheit und Radionuklidreinheit**			
2	**Bei zugelassenen Radiopharmazeutika die Fachinformation**			
3	**Bei nicht zugelassenen Radiopharmazeutika die Angaben zum Herstellungsverfahren und zur Qualitätskontrolle des Radiopharmazeutikums, die Namen der hierfür verantwortliche Personen sowie Angaben zu deren fachlichen Qualifikation**			

[1] SR **814.50**
[2] SR **814.501**

Nr.	Dokumentbezeichnung	Datum/ Versions-nummer	Allfälliger Ver-weis auf anderes Dokument	KEK: Bemerkung (freilassen)
4	**Die Namen der für die Anwendung des Radiopharma-zeutikums am Menschen verantwortlichen Personen sowie Angaben zu deren fachlichen Qualifikation**			
5	**Angaben gemäss Formular des BAG für Forschungspro-jekte mit Radiopharmazeutika oder mit radioaktiv markierten Stoffen[3]**			

Ethikkommission

Ort/Datum:

18. Nov. 2014

Fr. I. Oberli
Geschäftsführerin der Ethikkommission
Nordwest- und Zentralschweiz / EKNZ
Hebelstrasse 53
CH-4056 Basel

Wissenschaftliches Sekretariat

[3] Dieses Formular kann beim Bundesamt für Gesundheit, Abteilung Strahlenschutz, 3003 Bern, bezogen oder der Internetadresse www.bag.admin.ch >Themen>Strahlung, Radioaktivität und Schall>Nuklearmedizin und Forschung>Radiopharmazeutika>Gesuchsformular abgerufen werden.
