# Supplementary material for: Significant improvement of olfactory performance in sleep apnea patients after three months of nasal CPAP therapy – Observational study and randomized trial
Source: PLoS One. 2017 Feb 3;12(2):e0171087. doi: 10.1371/journal.pone.0171087 (PMC5291379; doi:10.1371/journal.pone.0171087)
Supplement: S6 File — Approval of amendment. (PDF) [file pone.0171087.s006.pdf]

11. Mai 2016

Die Ethikkommission Nordwest- und Zentralschweiz hat die vorliegenden Akten

[X] zur Kenntnis genommen

[X] genehmigt

Datum / Unterschrift

12.05.16

H1 / G1.287

Kantonsspital Aarau

Kantonsspital Aarau AG
Tellstrasse, CH-5001 Aarau
Tel. +41 (0)62 838 41 41
www.ksa.ch

Pneumologie und Schlafmedizin
PD Dr. med. S. Irani, Chefarzt
Dr. med. G. M. Tini, Leitender Arzt
Tel. Sekr. +41 (0)62 838 44 72
Fax +41 (0)62 838 44 69

Herr
Prof. Dr. med. André P. Perruchoud
Präsident der Ethikkommission Nordwest- und Zentralschweiz
Hebelstrasse 53
5056 Basel

Aarau, 10.05.2016/rh

Betreff: Amendment: EKNZ 2014-335: Change of olfactory performance after initiation of CPAP therapy in sleep apnea: a longitudinal study

Sehr geehrter Herr Professor Perruchoud

Oben genannte Studie wurde planmässig abgeschlossen. Wir konnten 35 Patienten in die Studie einschliessen und fanden drei Monate nach Beginn einer CPAP Therapie eine insgesamt signifikante, bei einigen Patienten massive Besserung des Geruchsinnes.

Um diese Daten noch weiter zu validieren möchten wir, falls der individuell angefragte Patient einverstanden ist eine Randomisierung durchführen und bei der Hälfte der Patienten die CPAP Therapie während drei Wochen auf subtherapeutische Werte reduzieren. Danach würden wir die Geruchstestung nochmals durchführen, was verblindet geschehen würde. Wir erwarten, dass jene Patienten, welche unter der etablierten CPAP Therapie bleiben sich gegenüber der letzten Geruchstestung wenig ändern, während jene Patienten unter sham-CPAP sich nach drei Wochen wieder verschlechtern. Das Prinzip der vorübergehenden sham-Bahandlung ist in der CPAP Forschung weit verbreitet [1-3].
Ich möchte Sie höflich anfragen, ob dieses Vorgehen im Sinne eines Amendment bewilligt würde. Die Untersuchungen würden im Zeitraum zwischen 1.6.2016 und 30.9.2016 durchgeführt.

Mit bestem Dank für Ihre wohlwollende Prüfung

PD Dr. med. S. Irani

(1) Strollo PJ, Jr., Soose RJ, Maurer JT, de VN, Cornelius J, et al. (2014) Upper-airway stimulation for obstructive sleep apnea. N Engl J Med 370: 139-149.

(2) Schwarz EI, Martinez-Lozano SP, Bregy L, Gaisl T, Garcia GD, et al. (2016) Effects of CPAP therapy withdrawal on exhaled breath pattern in obstructive sleep apnoea. Thorax 71: 110-117.

(3) Schwarz EI, Schlatzer C, Stehli J, Kaufmann PA, Bloch KE, et al. (2016) Effect of CPAP Withdrawal on myocardial perfusion in OSA: A randomized controlled trial. Respirology .

Beilagen:
- Patienteninformation vom Version 1 vom 04.05.2016
- Brief an Patient vom 03.05.2016
